# Respiratory Viral Testing Rate Patterns in Young Children Attending Tertiary Care Across Western Australia: A Population‐Based Birth Cohort Study

**DOI:** 10.1111/irv.70005

**Published:** 2024-09-03

**Authors:** Belaynew W. Taye, Mohinder Sarna, Huong Le, Avram Levy, Cara Minney‐Smith, Peter Richmond, Robert Menzies, Christopher C. Blyth, Hannah C. Moore

**Affiliations:** ^1^ Wesfarmers Centre of Vaccines and Infectious Diseases, Telethon Kids Institute University of Western Australia Perth Australia; ^2^ School of Population Health Curtin University Perth Australia; ^3^ Pathogen Genomics and Surveillance Unit, PathWest Laboratory Medicine QEII Medical Centre Perth Australia; ^4^ School of Biomedical Sciences University of Western Australia Perth Australia; ^5^ Department of Microbiology, PathWest Laboratory Medicine QEII Medical Centre Perth Australia; ^6^ School of Medicine University of Western Australia Perth Australia; ^7^ Department of Immunology Perth Children's Hospital Perth Australia; ^8^ Sanofi Vaccines Sanofi‐Aventis, Australia and New Zealand, Sydney Macquarie Park Australia

**Keywords:** Australia, geographic variation, respiratory virus, within‐hospital laboratory testing

## Abstract

**Background:**

An understanding of viral testing rates is crucial to accurately estimate the pathogen‐specific hospitalisation burden. We aimed to estimate the patterns of testing for respiratory syncytial virus (RSV), influenza virus, parainfluenza virus (PIV) and human metapneumovirus (hMPV) by geographical location, age and time in children <5 years old in Western Australia.

**Methods:**

We conducted a population‐based cohort study of children born between 1 January 2010 and 31 December 2021, utilising linked administrative data incorporating birth and death records, hospitalisations and respiratory viral surveillance testing records from state‐wide public pathology data. We examined within‐hospital testing rates using survival analysis techniques and identified independent predictors of testing using binary logistic regression.

**Results:**

Our dataset included 46,553 laboratory tests for RSV, influenza, PIV, or hMPV from 355,021 children (52.5% male). Testing rates declined in the metropolitan region over the study period (RSV testing in infants: from 242.11/1000 child‐years in 2012 to 155.47/1000 child‐years in 2018) and increased thereafter. Conversely, rates increased in non‐metropolitan areas (e.g., RSV in Goldfields: from 364.92 in 2012 to 504.37/1000 child‐years in 2021). The strongest predictors of testing were age <12 months (adjusted odds ratio [aOR] = 2.25, 95% CI 2.20–2.31), preterm birth (<32 weeks: aOR = 2.90, 95% CI 2.76–3.05) and remote residence (aOR = 0.77, 95% CI 0.73–0.81).

**Conclusion:**

These current testing rates highlight the potential underestimation of respiratory virus hospitalisations by routine surveillance and the need for estimation of the true burden of respiratory virus admissions.

## 1 Introduction

Acute lower respiratory infections (ALRIs) are the leading cause of hospitalisation and mortality in children, causing an estimated 45 million episodes of illness and 682,000 deaths globally in 2019 [1]. Respiratory syncytial virus (RSV) is the most prevalent cause of ALRIs in young children associated with 33 million ALRI episodes, 3.6 million hospitalisations and an estimated 101,400 deaths in children <5 years of age worldwide in 2019 [2]. Globally, influenza virus is associated with an estimated 10.1 million ALRIs, 870,000 hospital admissions and 34,800 deaths in children <5 years in 2018 [3]. Parainfluenza virus (PIV) (725,000 hospital admissions) [4] and human metapneumovirus (hMPV) (643,000 hospital admissions) [5] are significant contributors to child morbidity and health expenditure worldwide.

In Australia, the incidence of ALRIs is high, with an estimated 68.9 million cases reported annually. The highest burden is observed in children <5 five years [6]. In Western Australia (WA), ALRIs are associated with one in four emergency department presentations [7]. The incidence of respiratory virus‐associated ALRIs varies widely by age, seasonality, time and climate.

Variations in laboratory testing rates for respiratory viruses requiring hospitalisation may reflect differences in accessibility of laboratory tests, differences in laboratory capacity across different geographical regions and individual‐level socio‐economic factors. Respiratory virus testing has a substantial impact on the accurate estimation of the burden of respiratory virus‐related diseases. An optimal coverage of viral testing provides critical evidence to assist in prevention policy for interventions such as immunization, by providing a robust estimate of the pathogen‐specific burden by age and seasonality of respiratory viruses. However, as we have shown in the WA context, the true burden of respiratory viruses may be underestimated by 30%–57% [8].

An understanding of the testing patterns guides healthcare decision‐making to prevent respiratory virus‐associated hospitalisations. However, there is a scarcity of contemporary data exploring viral testing patterns and socio‐demographic predictors of testing in different geographical locations in WA. This study aimed to estimate and describe the testing rate patterns of RSV, influenza, PIV, and hMPV by health regions of WA, and time in children aged <5 five years.

## 2 Methods

### 2.1 Study Design

We conducted a whole‐of‐population‐based cohort study using probabilistically linked administrative data from WA children between January 2012 and December 2021.

### 2.2 Study Setting

WA covers the western third of Australia with a population of approximately 2.7 million; 77.8% of people live in the capital city, Perth and the surrounding metropolitan region [9]. There are 10 health regions of WA, three metropolitan health regions including Perth Metropolitan Region and seven WA regional areas namely, Kimberley, Pilbara, Midwest, Goldfields, Southwest, Wheatbelt and Great Southern (Figure S1). WA is characterised by a diverse climate with a tropical climate in the north and a temperate climate in the south.

### 2.3 Population

Our birth cohort included all children aged <5 years born in WA between 1 January 2010 and 31 December 2021. Children were followed from the date of birth until the earliest of 31 December 2021 (the date of censoring), when they turned 5 years of age, or the date of death.

### 2.4 Data Sources

**FIGURE 1:**
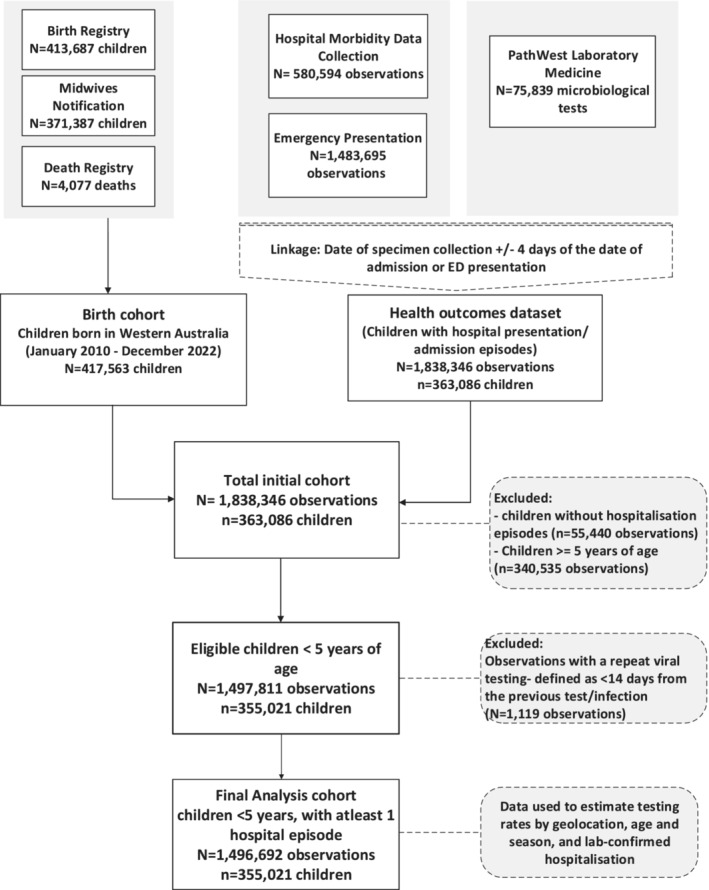
Data sources and study flowchart. The data sources presented in the figure are for the analysis to answer the main aim of the study. Data that were used to explain the interpretation of the study were presented in the supporting information. ED, emergency department.

Data for this study were obtained from the WA Respiratory Infections Linked Data Platform. The details on the construct of the cohort and linkage process of the platform are provided elsewhere [10]. In brief and of relevance for this analysis, the platform included demographic, hospitalisation and microbiological testing records from the Birth and Death Registry, Midwives Notification System, Hospital Morbidity Data Collection [HMDC], Emergency Department Data Collection [ED]), WA Notifiable Infectious Disease Database (WANIDD) and routine respiratory microbiological testing data from PathWest Laboratory Medicine (PathWest). Data were probabilistically linked by the WA Department of Health (Figure 1). Access to individually‐linked data was granted by the WA Department of Health Human Research Ethics Committee (RGS# 4675) and the WA Aboriginal Health Ethics Committee (Reference# HREC1138).

### 2.5 Morbidity Data

We included all paediatric hospital admissions in WA between 1 January 2012 and 31 December 2021 for children in the cohort. Duplicate records from the HMDC dataset by child's unique identification number, date of admission and date of discharge were identified and removed. Where there were different discharge dates but the same admission dates for a child, we retained the observation with the most recent discharge date. We retained primary diagnoses attached to a duplicate record if the diagnoses were different from the primary record. All inter‐hospital transfers, from the HMDC dataset, were collapsed. All emergency presentations for children <5 years of age to all hospitals in WA between 2012 and 2021 were included in the cohort. Hospital admissions (hereafter referred to as hospitalisations) and emergency department presentations were combined to form the health outcome data for the cohort (Figure 1).

### 2.6 Microbiological Data

Microbiological testing records for respiratory viruses were obtained from PathWest. PathWest is the WA's sole public pathology provider, with more than 200 laboratories and collection centres providing a substantial coverage of laboratory services in the metropolitan and regional areas of WA [11]. Laboratory data including routine respiratory viral testing (positive and negative results) using PCR on respiratory samples from 1 January 2012 to 31 December 2021. PathWest data were linked with the health outcome records where the date of specimen collection was 4 days either side of the date of admission or ED presentation [10]. A consecutive laboratory test for a respiratory virus was defined as a PCR test performed for the same child after 14 days of the previous test—the time where shedding of the previous infection is expected to stop [12, 13]. To avoid double‐counting of tests and laboratory confirmed hospital presentations, we excluded repeat testing from this analysis.

To determine the rates of respiratory virus testing, we identified the number of admissions/ED presentations that were temporally linked to laboratory testing for RSV, influenza, PIV and hMPV, allowing a child to have multiple tests throughout the follow‐up period.

### 2.7 Clinical and Sociodemographic Variables

The primary outcome variable was testing for respiratory virus (coded as tested or not tested). To generate the composite outcome variable and identify predictors of testing, all viruses RSV, influenza, PIV and/or hMPV were grouped together and testing for a respiratory virus was defined as having had a test for either of the four viruses. The date of specimen collection was used in determining the date of the test.

We described the patterns of respiratory viral testing by WA regions, age at the time of hospitalisation (months), season of admission and year of testing. Health service regions were classified using postcode of residence at the time of health outcome, which in turn were obtained from the list of postcodes included in the catchment areas for each health service region available from the WA Department of Health [14]. The season of admission was computed from the date of admission or the date of specimen collection and grouped as summer (December to February), autumn (March to May), winter (June to August) and spring (September to November). We explored testing rates by age at the time of health outcome and grouped the age at the time of hospitalisation into <12 months, 12–23 months and 24–59 months.

The statistical area from the Australian Census 2011 and 2016 reports was used to define remoteness and socioeconomic index for areas. Due to the possibility of relocation within WA from birth to the time of a health outcome, we used the geolocation data from the closest census period using the date of specimen collection as a reference. We used the remoteness of areas and socio‐economic advantage and disadvantage index values for the Statistical Area Level 1 geolocation from the 2011 census for observations whose date of admission was on or before 31 December 2013, and Statistical Area Level 1 geolocation from the 2016 census data for admissions between 1 January 2014 and 31 December 2021. The remoteness of residence was measured using the Accessibility/Remoteness Index of Australia (ARIA), which is an index of scores based on the distance from localities to population centres. The remoteness of areas is divided into major cities, inner regional, outer regional, remote and very remote based on a measure of relative access to services [15]. The socio‐economic indexes for areas (SEIFA) was classified according to the relative socio‐economic advantage and disadvantage of localities using Census data, and this ranges from most disadvantaged (Decile 1) to most advantaged (Decile 10) [16].

### 2.8 Statistical Analysis

Respiratory virus testing rates were calculated using survival analysis methods allowing multiple testing for a child over time, and we used child‐years of observation as a denominator. Rates were computed for hospitalisations and ED presentations combined. We explored proportions for International Classification of Disease (ICD)‐coded admissions for acute lower respiratory illnesses including bronchiolitis, viral pneumonia and bronchitis. Initially, missing data were checked, and percentages were examined, and any variable with missing values more than 10% was excluded from analysis. The child‐years of follow‐up were calculated from the date of birth, admission date, discharge date and date of censoring. We reported testing rates per 1000 child‐years with corresponding 95% confidence intervals.

Independent predictors of testing for respiratory virus (defined as testing for either RSV, influenza, PIV, or hMPV) were identified using multivariable binary logistic regression. Initially, a bivariable logistic regression was performed for gestational age at birth, age at admission, sex, ethnicity, maternal age, maternal smoking during pregnancy, presence of multiple births, maternal asthma diagnosis, asthma diagnosis in the child, febrile and other viral illnesses, remoteness of residence, WA Health region, season of testing and year of testing. Variables with a *P*‐value less than 0.2 in the bivariable regression analysis were included in the multivariable logistic regression. Odds ratios with 95% confidence intervals were reported to compare the difference in the odds of testing between categories of predictor variables and to determine statistical significance. Multicollinearity between predictor variables was assessed by computing the variance inflation factor using collinearity diagnostics. Due to seasonal variation among regions of WA, we used interaction term between season of admission and region of residence, and the best fitting model was identified using likelihood ratio test. Pearson's test was used to assess the goodness of fit of the binary logistic regression model.

## 3 Results

A total of 355,021 children (52.5% males) born between 1 January 2010 and 31 December 2022 and aged <5 years at the time of hospitalisation/ED presentation (Figure 1) were included in the analysis. Nearly two‐thirds (*n* = 220,823) of children were admitted to hospital, and 134,198 children presented to ED at their first presentation. The majority of children (*n* = 277,339, 78.1%) were from metropolitan regions of WA. At the time of hospitalisation/ED presentation, nearly one‐in‐ten children lived in remote (*n* = 15,343, 4.7%) or very remote (*n* = 8019, 2.5%) areas (Table S1).

There were 46,553 laboratory tests for either of the four respiratory viruses studied: RSV (*n* = 45,601), influenza (*n* = 46,164), PIV (*n* = 43,123) and hMPV (*n* = 43,322) throughout the study period. For infants <12 months in WA, the overall testing rate for the period 2012–2021 for RSV was 256.02, influenza was 258.87, PIV was 247.01, and hMPV was 248.00 per 1000 child‐years.

### 3.1 Patterns of Respiratory Virus Testing Rates by Geographical Location

Table 1 presents the patterns of testing for RSV, influenza, PIV and hMPV by age subgroup and location. In the Perth metropolitan region, RSV testing rate in children <12 months was 207.36/1000 child‐years and 194.20/1000 for PIV. For children 24–59 months of age, the testing rates were 18.98/1000 for RSV and 17.63/1000 child‐years for PIV.

**TABLE 1:** Patterns of testing for respiratory viruses in children <5 years of age across Western Australia, 2012–2021.

Region	Age of child					
	<12 months		12–23 months		24–59 months	
	Number tested	Rate^a^ (95% CI)	Number tested	Rate (95% CI)	Number tested	Rate (95% CI)
Respiratory syncytial virus						
Perth metropolitan	14,453	207.36 (203.57–211.24)	7368	53.20 (51.76–54.69)	7146	18.98 (18.36–19.63)
Southwest	1109	165.64 (155.26–176.90)	546	41.13 (37.39–45.35)	584	15.16 (13.76–16.74)
Kimberley	1112	325.61 (304.44–348.66)	645	135.88 (124.34–148.80)	585	48.60 (44.16–53.62)
Pilbara	1067	259.96 (241.89–279.74)	574	87.11 (79.58–95.55)	577	35.19 (32.16–38.60)
Wheatbelt	487	169.20 (151.84–189.12)	210	38.20 (32.45–45.28)	239	16.06 (12.94–20.20)
Goldfields	1476	445.58 (418.76–474.56)	643	115.23 (105.43–126.19)	547	40.94 (36.81–45.67)
Midwest	943	267.52 (248.42–288.49)	446	75.54 (67.57–84.73)	442	27.18 (24.48–30.26)
Great Southern	495	207.31 (188.34–228.73)	290	63.74 (55.90–73.02)	309	24.76 (21.52–28.64)
Influenza virus						
Perth metropolitan	14,682	210.64 (206.82–214.56)	7457	53.84–52.39–55.34)	7251	19.26 (18.63–19.92)
Southwest	1121	167.43 (156.96–178.79)	549	41.35 (37.60–45.59)	591	15.34 (13.94–16.93)
Kimberley	1138	333.22 (311.64–356.71)	654	137.78 (126.10–150.85)	590	49.02 (44.53–54.08)
Pilbara	1068	260.20 (242.11–280.01)	577	87.56 (79.98–96.07)	577	35.19 (32.16–38.60)
Wheatbelt	499	173.37 (155.81–193.48)	214	38.92 (32.97–46.29)	240	16.13 (13.00–20.27)
Goldfields	1485	448.29 (421.39–477.36)	645	115.59 (105.77–126.57)	555	41.54 (37.31–46.39)
Midwest	949	269.23 (249.96–290.38)	447	75.71 (67.74–84.90)	443	27.24 (24.53–30.33)
Great Southern	498	208.57 (189.47–230.14)	291	63.96 (56.11–73.25)	311	24.92 (21.68–28.81)
Parainfluenza virus						
Perth metropolitan	13,536	194.20 (190.53–197.96)	6780	48.95 (47.58–50.38)	6638	17.63 (17.03–18.27)
Southwest	1069	159.66 (149.51–170.69)	522	39.32 (35.66–43.45)	564	14.64 (13.27–16.20)
Kimberley	1068	312.73 (292.05–335.27)	626	131.88 (120.50–144.64)	575	47.77 (43.35–52.76)
Pilbara	1027	250.21 (232.63–269.49)	561	85.14 (77.71–93.46)	563	34.34 (31.34–37.71)
Wheatbelt	469	162.95 (145.87–182.60)	201	36.56 (30.91–43.57)	228	15.32 (12.25–19.44)
Goldfields	1452	438.33 (411.77–467.05)	634	113.62 (103.88–124.52)	531	39.74 (35.67–44.42)
Midwest	916	259.86 (241.07–280.53)	429	72.66 (64.83–81.72)	421	25.89 (23.27–28.89)
Great Southern	473	198.10 (179.60–219.04)	281	61.77 (54.05–70.91)	300	24.04 (20.86–27.86)
Human metapneumovirus						
Perth metropolitan	13,586	194.92 (191.24–198.69)	6754	48.77 (47.39–50.19)	6639	17.64 (17.03–18.27)
Southwest	1072	160.11 (149.95–171.15)	524	39.47 (35.80–43.62)	563	14.62 (13.24–16.18)
Kimberley	1070	313.31 (292.59–335.90)	627	132.09 (120.70–144.86)	575	47.77 (43.35–52.76)
Pilbara	1027	250.21 (232.63–269.49)	562	85.29 (77.83–93.66)	563	34.34 (31.34–37.71)
Wheatbelt	475	165.03 (147.93–184.69)	201	36.56 (30.74–43.83)	227	15.26 (12.18–19.37)
Goldfields	1454	438.94 (412.37–467.66)	634	113.62 (103.88–124.52)	534	39.97 (35.83–44.72)
Midwest	917	260.15 (241.35–280.82)	429	72.66 (64.86–81.69)	420	25.82 (23.21–28.82)
Great Southern	472	197.68 (179.25–218.54)	281	61.77 (54.05–70.91)	300	24.04 (20.86–27.86)

^a^ Per 1000 child–years.

In the regional areas of WA, respiratory virus testing rates were the highest for all age groups in the Goldfields (e.g., 445.58/1000 for RSV and 438.94/1000 child‐years for hMPV in infants) followed by Kimberley (e.g., 325.61/1000 for RSV and 313.31/1000 for hMPV in infants). Testing rates for all viruses were the lowest in the Southwest (e.g., 165.64/1000 child‐years for RSV and 160.11/1000 for hMPV in infants) and Wheatbelt (e.g., 169.20/1000 child‐years for RSV and 165.03/1000 for hMPV for the same age group) regions (Table 1).

### 3.2 Patterns of Respiratory Virus Testing by Age and Time

**FIGURE 2:**
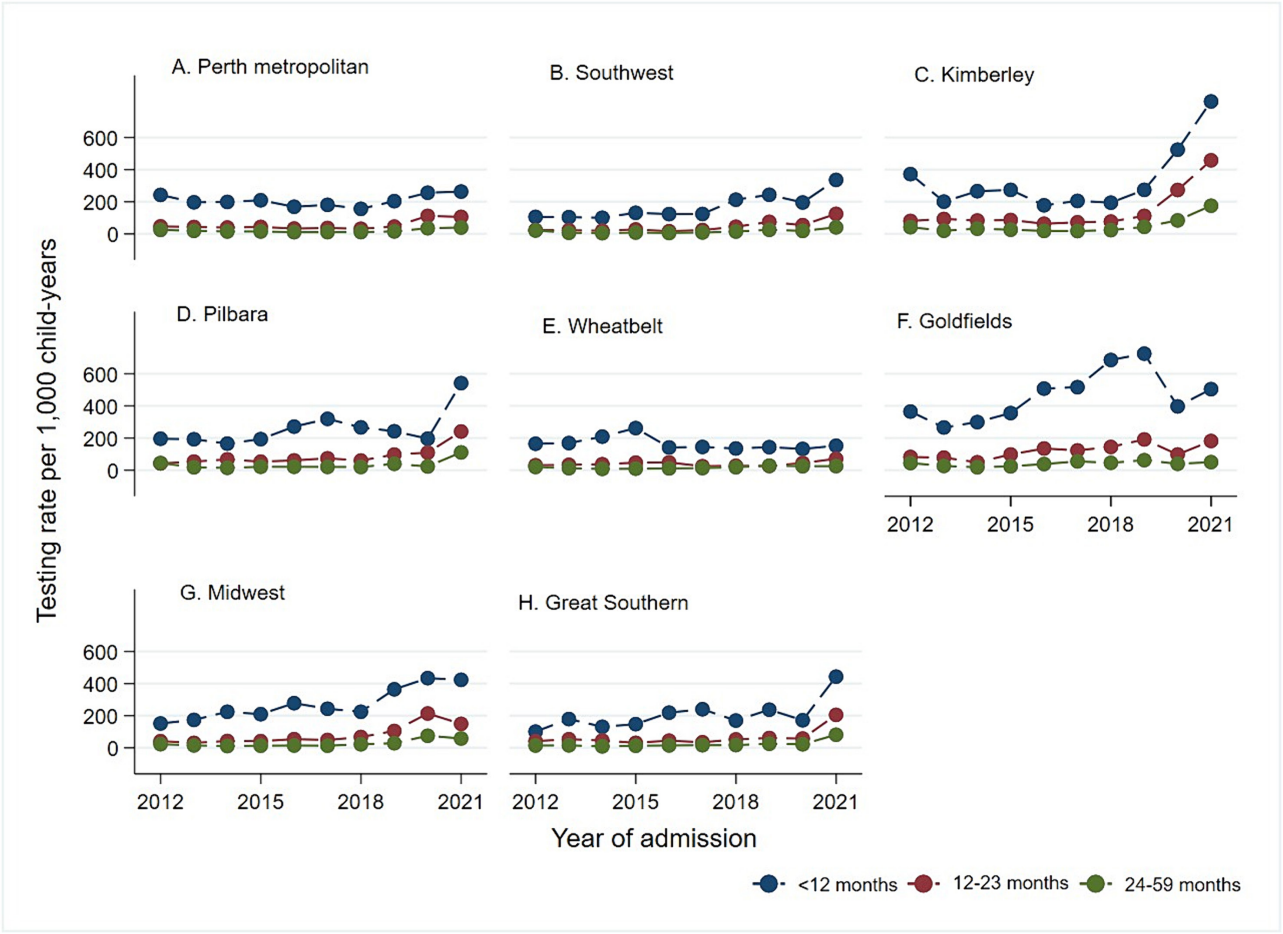
Patterns of respiratory syncytial virus testing rate by age and time across regions of Western Australia. The lines present patterns of testing rates for children <12 months (blue), children 12–23 months of age (red), and children 24–59 months of age (green) in Perth metropolitan (A), Southwest (B), Kimberley (C), Pilbara (D), Wheatbelt (E), Goldfields (F), Midwest (G), and Great Southern (H).

Figure 2 and Figures S2–S4 highlight the patterns of testing for RSV, influenza, PIV and hMPV by age and year of admission for children who presented to ED or were hospitalised throughout WA. Testing rates for RSV, influenza, PIV and hMPV were widely variable by age, with higher rates for children <12 months of age for all four viruses. In the Perth metropolitan region in 2012, the testing rate for RSV was 242.11/1000 child‐years in infants compared with 45.99/1000 child‐years in the 12‐ to 23‐month‐olds and 24.46/1000 child‐years in the 24‐ to 59‐month‐olds (Figure 2, Table S2). Similarly, in the Perth metropolitan region, testing rates were higher for influenza (251.07 vs. 26.13/1000 child‐years), PIV (241.35 vs. 24.31/1000 child‐years) and hMPV (247.12 vs. 24.92/1000 child‐years) in infants compared with children aged 24–59 months (Tables S3–S5). In WA regional areas, a higher testing rate in children <12 months of age compared with children 24–59 months, was found for all the four viruses (e.g., 345.13 vs. 36.93/1000 child‐years for hMPV in the Kimberley region) (Figures S2–S4).

There were variable patterns of testing rates for all four respiratory viruses by time and by WA Health regions. In the Perth metropolitan region, we found a decreasing pattern of respiratory virus testing rate from 2012 to 2018 (RSV: 242 vs. 155/1000 child‐years) and followed by an increase in the rate from 2019 onwards to 2021 where the testing rate for RSV was 263/1000 child‐years in infants (Figure 2). A similar decreasing pattern of testing by year was observed for influenza, PIV and hMPV (Figure S2–S4). Conversely, in the WA regional areas, there was an increasing trend in testing rates for respiratory viruses by time across all age groups. In the Goldfields region, for example, the testing rate for RSV in children <12 months increased from 365/1000 child‐years in 2012 to 504/1000 child‐years in 2021. In the tropical Kimberley region, RSV testing rate was 372/1000 in 2012 and increased to 824/1000 child‐years in 2021 (Figure 2). Similarly, testing rates in all age groups for influenza, PIV and hMPV increased between 2012 and 2021 across all regional areas of WA. Further details are provided in Figures S2–S4.

Using influenza virus notifications data from the WA Notifiable Infectious Diseases Database (which includes notifications from all public and private laboratories across the state) linked to the WA Respiratory Infections Linked Data Platform, we examined the contribution of microbiological testing from non‐PathWest testing sources to understand the contribution of testing in the private laboratories (Figure S5). While the number of influenza‐confirmed hospitalisations was similar between those recorded in the notifiable disease register and tested within the PathWest to those tested outside of PathWest in 2015–2017, an increase in influenza‐confirmed hospitalisations tested outside of PathWest was seen in 2018, and in particular in 2019. An additional 48 cases in 2018 and 206 cases in 2019 were identified that were not captured in the PathWest data, alluding to a significant contribution of the private sector towards the tests conducted for common respiratory viruses.

### 3.3 Respiratory Virus Test Positivity Rate

Respiratory viral positivity rates by age are shown in Table S6. RSV positivity rates were high for infants compared with the positivity rates in older age groups (e.g., 26.15 vs. 15.05% compared with children 24‐ to 59‐month‐olds for the Perth metropolitan region). In contrast, positivity rates for influenza (1.81 vs. 6.63%), PIV (5.47 vs. 5.87%) and hMPV (4.09 vs. 5.73%) were lower in infants compared with the positivity rates in pre‐school children 24–59 months of age.

Figure S6 presents the respiratory virus positivity rate by geographical location for children <5 years of age tested for RSV, influenza virus, PIV and hMPV. Across all the WA geographic regions, the positivity rate was markedly higher for RSV compared with the rates for the other viruses. For example, in 2012 in the Perth metropolitan region, RSV positivity rate was 25.35%, higher than the positivity rates for PIV (4.28%), hMPV (3.07%) and influenza (3.09%).

### 3.4 Laboratory‐Confirmed RSV Hospitalisations for Acute Respiratory Illnesses

**FIGURE 3:**
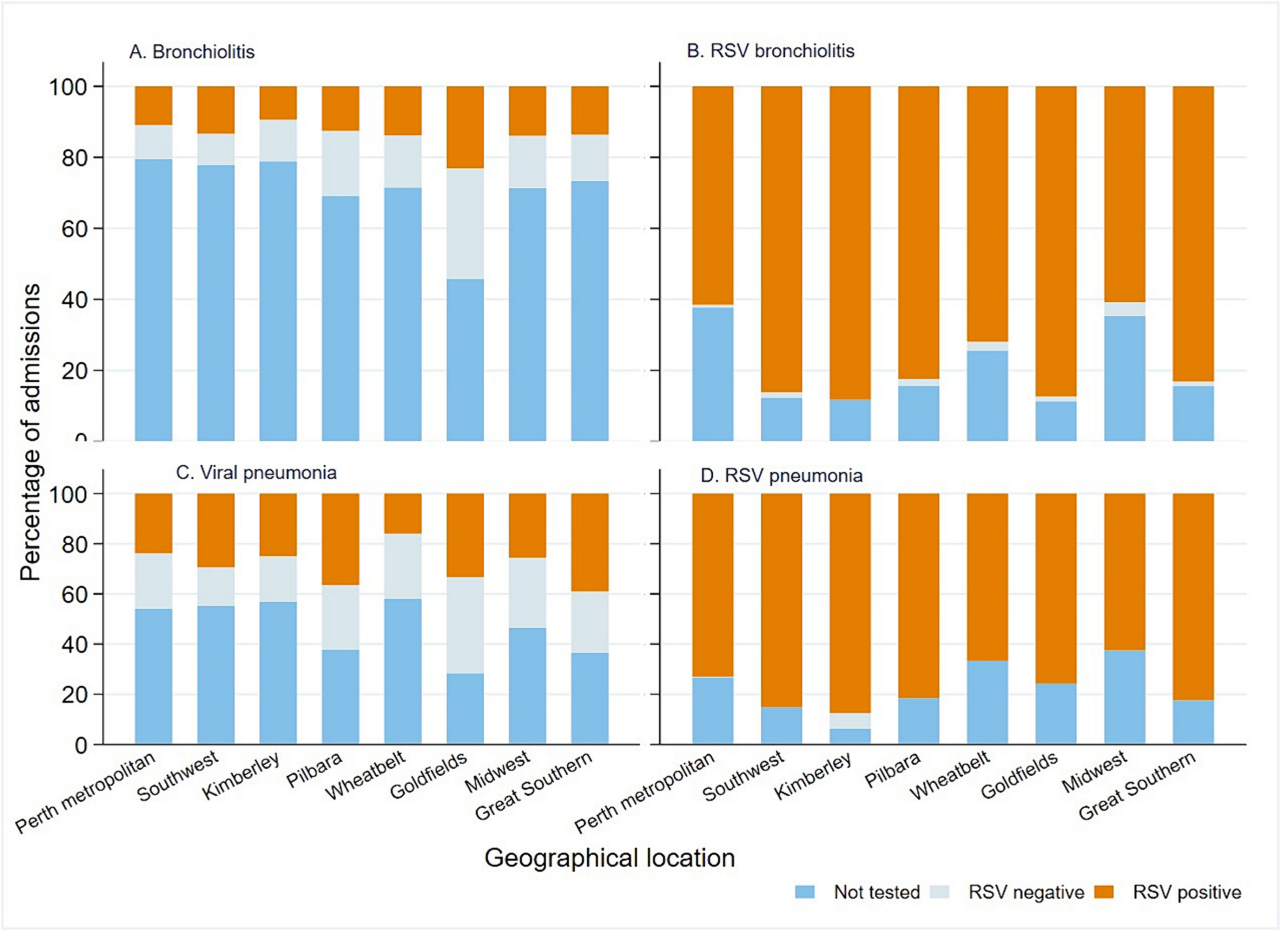
Proportion of laboratory‐confirmed RSV hospitalisations for ICD‐coded admissions in children <5 years in the pre‐COVID era (2012–2019). The figures represent ICD‐coded hospitalisations/ED presentations including (A) bronchiolitis, (B) RSV bronchiolitis, (C) viral pneumonia, (D) RSV pneumonia. ICD, International Classification of Disease; RSV, respiratory syncytial virus.

Figures 3 and S7 present the testing and positivity rates of RSV for selected ICD‐coded hospitalisations, including general ALRI codes such as bronchiolitis (J21) and viral pneumonia (J12), and RSV‐specific codes such as RSV bronchiolitis (J21.0), and RSV pneumonia (J12.1) in children <5 years of age in the pre‐COVID (2012–2019) and COVID epidemic (2020–2021) periods. Not all admissions with RSV‐specific codes for bronchiolitis or pneumonia had evidence of a laboratory test for RSV, and approximately 10%–58% of the non‐specific admission codes were tested and found positive for RSV. During the pre‐COVID epidemic period (Figure 3), RSV testing rate for children hospitalised with a primary diagnosis code of bronchiolitis was lower than the rate in the COVID era (20.0 vs. 25.0% in the Perth metropolitan region) (Figure S7). Overall, the proportion of laboratory‐confirmed RSV hospitalisations for the primary diagnosis of viral pneumonia ranged from 16% in the Wheatbelt to 22% in Perth metropolitan, and 36% in the Pilbara regions during the pre‐COVID period.

### 3.5 Predictors of Testing for Respiratory Virus

In a multiple logistic regression analysis, respiratory virus testing was strongly predicted by age of the child at presentation, gestational age, remoteness of residence, geographical region, admission with an ICD‐coded diagnosis of asthma and presentation with any febrile illness (Table 2). Children aged <12 months at the time of admission to hospital/ED presentation had more than two times [adjusted odds ratio (aOR) = 2.25, 95% CI 2.20–2.31] higher odds of being tested for respiratory viruses compared with 24‐ to 59‐month‐old children. Living in a remote or very remote location at the time of admission decreased the odds of being tested by 23% (aOR = 0.77, 95% CI 0.73–0.81). Children in the Wheatbelt region had 40% (aOR = 0.61, 95% CI 0.51–0.72) lower adjusted odds of testing for respiratory viruses compared with those residing in Perth metropolitan area and having had a primary diagnosis of asthma increased the odds of testing by nearly four times (aOR = 3.95, 95% CI 3.59–4.35) (Table 2).

**TABLE 2:** Predictors of within‐hospital testing for respiratory virus in children <5 years of age across Western Australia.

Predictor variable	Testing for respiratory virus	
	Unadjusted odds ratio (95% CI)	Adjusted odds ratio (95% CI)
Age of child (ref: 24–59 months)		
<12 months	2.07 (2.02–2.12)	2.25 (2.20–2.31)
12–23 months	1.68 (1.63–1.72)	1.75 (1.70–1.80)
Sex (ref: female)		
Male	1.09 (1.06–1.11)	1.08 (1.06–1.10)
Gestational age at birth (ref: 37+ weeks)		
<32 weeks	3.25 (3.11–3.40)	2.90 (2.76–3.05)
32–36 weeks	1.46 (1.42–1.50)	1.35 (1.31–1.39
Remoteness of residence (ref: major city)		
Regional	1.01 (0.98–1.03)	1.06 (1.01–1.11)
Remote/very remote	0.97 (0.94–0.99)	0.77 (0.73–0.81)
Socioeconomic Index for Areas (SEIFA)	1.004 (0.001–1.01)	1.01 (1.003–1.011)
Season of admission (ref: summer)		
Autumn (March–May)	1.11 (1.08–1.15)	1.17 (1.13–1.21)
Winter (June–August)	1.78 (1.73–1.83)	1.76 (1.70–1.82)
Spring (September–November)	1.33 (1.29–1.36)	1.21 (1.17–1.25)
Maternal age (ref: <20 years)		
20–24 years	1.01 (0.96–1.06)	1.05 (0.99–1.10)
25–29 years	1.00 (0.95–1.04)	1.06 (1.00–1.11)
30–34 years	1.05 (1.00–1.10)	1.11 (1.05–1.17)
35 year and above	1.11 (1.06–1.16)	1.16 (1.10–1.22)
Maternal smoking during pregnancy (ref: no)	1.14 (1.11–1.17)	1.08 (1.04–1.11)
Maternal asthma diagnosis (ref: no)	1.06 (1.03–1.09)	1.07 (1.04–1.10)
Ethnicity (ref: non‐Aboriginal)		
Aboriginal or Torres Strait Islander	1.25 (1.22–1.28)	1.23 (1.19–1.28)
Geographical location (ref: Perth metropolitan)		
Southwest	0.71 (0.58–0.74)	0.66 (0.59–0.74)
Kimberley	1.06 (1.02–1.11)	1.59 (1.43–1.76)
Pilbara	1.02 (0.98–1.07)	1.36 (1.22–1.52)
Wheatbelt	0.71 (0.67–0.75)	0.60 (0.50–0.72)
Goldfields	1.82 (1.75–1.89)	1.87 (1.68–2.09)
Midwest	1.03 (0.98–1.07)	1.10 (0.97–1.24)
Great Southern	1.00 (0.94–1.06)	1.27 (1.10–1.46)
Year of testing	1.05 (1.05–1.06)	1.10 (1.09–1.11)
Mode of delivery (ref: spontaneous delivery)		
Induced	0.98 (0.96–0.99)	0.97 (0.95–1.00)
Caesarean section	1.19 (1.16–1.22)	1.09 (1.06–1.12)
Plurality of birth (ref: singleton)		
Multiple birth	1.53 (1.46–1.60)	1.05 (1.00–1.11)
Child asthma diagnosis	2.82 (2.57–3.09)	3.95 (3.59–4.35)
Fever or other viral illnesses	1.59 (1.55–1.64)	1.52 (1.47–1.56)
Season + geographical location		
Autumn*Southwest	0.72 (0.65–0.79)	0.98 (0.84–1.14)
Autumn*Kimberley	1.34 (1.23–1.46)	0.89 (0.79–1.01)
Autumn*Pilbara	1.23 (1.12–1.34)	0.93 (0.81–1.07)
Autumn*Wheatbelt	0.75 (0.65–0.86)	1.20 (0.95–1.51)
Autumn*Goldfields	2.02 (1.85–2.20)	1.02 (0.89–1.17)
Autumn*Midwest	1.12 (1.01–1.24)	0.97 (0.83–1.14)
Autumn*Great Southern	1.09 (0.96–1.25)	0.83 (0.69–1.01)
Winter*Southwest	1.46 (1.36–1.56)	1.27 (1.11–1.44)
Winter*Kimberley	1.48 (1.36–1.61)	0.64 (0.57–0.72)
Winter*Pilbara	1.60 (1.48–1.73)	0.79 (0.69–0.90)
Winter*Wheatbelt	1.36 (1.23–1.50)	1.31 (1.07–1.61)
Winter*Goldfields	3.17 (2.96–3.40)	1.03 (0.91–1.17)
Winter*Midwest	1.82 (1.67–1.97)	1.01 (0.87–1.17)
Winter*Great Southern	1.61 (1.45–1.79)	0.75 (0.63–0.89)
Spring*Southwest	0.92 (0.84–1.00)	1.09 (0.94–1.26)
Spring*Kimberley	1.48 (1.36–1.61)	0.84 (0.74–0.95)
Spring*Pilbara	1.43 (1.31–1.56)	0.94 (0.82–1.08)
Spring*Wheatbelt	1.03 (0.92–1.16)	1.49 (1.20–1.85)
Spring*Goldfields	2.69 (2.50–2.90)	1.23 (1.08–1.40)
Spring*Midwest	1.52 (1.39–1.66)	1.24 (1.07–1.44)
Spring*Great Southern	1.36 (1.22–1.53)	0.85 (0.71–1.02)

## 4 Discussion

This cohort study explored the patterns of testing for RSV, PIV, influenza and hMPV in young children across various age subgroups, years and geographical locations in WA using a population‐based birth cohort of children.

Respiratory viral testing in the hospital setting is essential to understand the pathogen‐specific hospitalisation burden in the context of variability of testing practices. Estimates of within‐hospital viral testing patterns provide valuable information regarding the magnitude of underestimation of respiratory virus‐associated hospitalisation burden [8]. Consistent viral testing in the hospital setting, which would lead to timely initiation of treatment, has a substantial contribution to the prevention of respiratory virus‐associated intensive care unit admissions and mortality [17] and enhances efficient use of hospital resources [18]. Furthermore, accurate estimates of pathogen‐specific testing rates provide evidence to support preventive and therapeutic interventions, such as immunisation programs targeting RSV [19], which have begun across WA in April 2024.

Our study highlights a higher testing rate in infants compared with older children across all geographical regions and over time, with more than two times higher odds of testing compared with children 24–59 months of age. The higher viral testing rate in infants may be associated with the developing immunity against respiratory viruses on top of the waning passive immunity at a younger age. An undeveloped immunity in infants might be associated with a higher risk of reinfection over different seasons leading to a high incidence of respiratory virus infection and increased chance of being tested [6, 20]. Younger children may also be likely to have been admitted to the hospital/ED with a more severe clinical presentation of ALRIs [21], which might increase the likelihood of the treating physician recommending a PCR test for respiratory viruses [20, 22].

The testing rates between RSV, influenza, PIV and hMPV were similar due to the utilization of multipanel polymerase chain reaction testing, where testing one of the respiratory viruses increases the likelihood of a child being tested for the other viruses at the same time [23].

The respiratory virus testing rates within regional areas of WA were highly variable, with higher testing rates in the central Goldfields and northern Kimberley regions. Higher rates of testing in these regions might be associated with a high burden of respiratory virus infection in children, resulting in ED presentation or hospital admission with ALRIs, which is associated with an increased chance of being tested for respiratory viruses [7]. Moreover, in regional areas, children might present late with a more severe form of disease, and are thus more likely to get tested [7]. On the other hand, lower rates of respiratory virus testing in the Southwest and Great Southern regions reflects the varying testing practices in different geographical locations [24] which could be dependent on differing availability of staff, testing resources and hospital guidelines.

Recently, Australia has launched guidelines for the management of bronchiolitis, which does not recommend routine virological testing for children presenting to ED or admitted to hospital with a clinical diagnosis of bronchiolitis [25]. This recommendation might have contributed to a decrease in testing practices for respiratory viruses in implementing hospitals; however, there is likely to be variations in their implementation of the guidelines, evidenced by testing variability across WA. Paediatric respiratory viral testing may also be associated with the number of paediatric specialists practising in hospitals providing infectious disease diagnosis and treatment services for the catchment population.

Differing testing patterns over time were observed between metropolitan and regional areas of WA. Testing rates decreased between 2012 and 2018 in the Perth metropolitan region, while there was an increasing trend in testing for respiratory viruses in the same period in the WA regional areas. Decreasing trends of testing in the metropolitan region of WA, unlike previous reports [26], may be associated with an increasing contribution of the private laboratory service sector in the Perth metropolitan region. Using influenza notifications from the WA Notifiable Infectious Disease Data, we found an increase in the number of laboratory‐confirmed influenza hospitalisations reported from private testing sources (Figure S5), resulting in an apparent reduction in testing rates in the metropolitan areas. The increasing numbers for influenza testing from private laboratory may indicate that there is an expanding contribution of the private sector testing for respiratory viruses. The increasing trend in testing rate for respiratory viruses in regional areas of WA may be associated with the limited expansion of private laboratory testing in regional areas, and with PathWest remaining the most predominant respiratory virus testing provider.

An increase in viral testing rate in the metropolitan areas of WA starting from 2019 following a decreasing trend is most likely associated with the upsurge in global awareness of and attention to respiratory viruses after the onset of the COVID‐19 pandemic in 2020, leading to increased testing for all respiratory viruses [27]. A surge in testing rates following a sharp drop in 2020 may be explained by a decrease in the incidence of respiratory virus infections due to the impact of the implementation of public health measures to control the COVID‐19 epidemic [28, 29, 30, 31]. These measures resulted in reduced transmission of respiratory viruses and a resultant reduction in ALRI‐related ED presentations/hospital admissions [31, 32, 33]. This was followed by increasing testing rates for all the four viruses in 2021, in part due to the relaxing of social distancing restrictions associated with increased transmission [30, 34].

A higher positivity rate for RSV compared with other respiratory viruses across all health service regions of WA demonstrates that RSV is the most prevalent cause of ALRI hospitalisations in children <5 years old compared with other respiratory viruses [35]. When the positivity rates were examined by age, higher rates were observed for RSV in children <12 months of age, in contrast with rates for influenza and PIV, which were higher in children 24–59 months of age. The varying positivity rate across age subgroups reflects the differing epidemiology of respiratory viruses between infants and older children [19, 36, 37]. Our study highlights a higher RSV positivity rate for children admitted with ICD‐coded diagnoses of acute bronchiolitis, and viral pneumonia compared with children admitted for other diseases.

Remoteness of residence was a significant predictor of testing for a respiratory virus, with children residing in remote or very remote areas at the time of admission having had 23% less odds of being tested for respiratory viruses. Although testing rates have generally increased throughout the study period in regional areas of WA, in people residing in remote locations, testing for respiratory viruses could be impacted by the limited access to and use of tests compared with patients from metropolitan areas [38]. This indicates a disparity between the burden of respiratory viral illness and the access and utilization of health services including testing.

Although this study highlighted the patterns of respiratory virus testing in detail by geographical location, age and time using population‐based data, it has some limitations. First, while our birth cohort dataset included data up to 31 December 2022, laboratory testing data for PathWest were available only until 31 December 2021, which limited us from describing fully the post‐pandemic testing patterns and comparing it with the testing pattern during the pre‐pandemic years. Second, while we used influenza notification data to estimate the demonstrated an increasing contribution of the private testing providers to respiratory virus testing, our analysis included data only from PathWest, which is a public laboratory testing service. Although PathWest still provides a significant coverage of respiratory virus testing across WA, the absence of data from the private laboratory sector has limited our ability to describe the complete statewide picture of the testing patterns over time.

## 5 Conclusions

Decreasing testing rate between 2012 and 2018 in the Perth metropolitan region, may indicate the increasing share of the private laboratory testing sector in the metropolitan areas. Increasing testing rate patterns in the WA regional areas highlights a high burden of respiratory virus infection and more limited expansion of the private laboratory service sector in regional areas. The lower odds of testing for respiratory virus in children living in remote areas highlight the importance of striking the balance between disease burden, implementation of testing guidelines and access to testing services including availability of paediatricians.

The current testing rate for children presenting for tertiary care highlights an underestimation of the true burden of respiratory virus‐associated hospitalisations by routine surveillance. To provide robust evidence to guide ongoing prevention policy, especially RSV immunisation, further statistical prediction models across varying geographical regions are needed to estimate the true burden of respiratory viruses in children admitted for tertiary care.

## Author Contributions

**Belaynew Taye W:** conceptualization, methodology, data curation, validation, formal analysis, visualization, writing – original draft, writing – review and editing. **Mohinder Sarna:** data curation, writing – review and editing. **Huong Le:** data curation, writing – review and editing. **Avram Levy:** writing – review and editing. **Cara Minney–Smith:** writing – review and editing. **Peter Richmond:** writing – review and editing. **Robert Menzies:** writing – review and editing. **Christopher Blyth C:** writing – review and editing. **Hannah Moore C:** conceptualization, methodology, validation, formal analysis, funding acquisition, project administration, resources, writing – review and editing.

## Conflicts of Interest

The authors declare no conflicts of interest.

## Ethics Statement

This study was approved by the WA Health Human Research Ethics Committee (HREC RGS Reference: #4675).

## Supporting information


**Table S1.** Characteristics of the cohort.
**Table S2.** Respiratory syncytial virus testing rate patterns by age and health regions in Western Australia, 2012–2021.
**Table S3.** Influenza virus testing rate patterns by age and health service regions in Western Australia, 2012–2021.
**Table S4.** Parainfluenza virus testing rate patterns by age and health service regions in Western Australia, 2012–2021.
**Table S5.** Human metapneumovirus testing rate patterns by age and health regions in Western Australia, 2012–2021.
**Table S6.** Respiratory virus positivity rates by age across Western Australia, 2012–2021.
**Figure S1.** Map of Western Australia health regions.
**Figure S2.** Patterns of influenza testing rates by age across health service regions of Western Australia.
**Figure S3.** Patterns of PIV testing rates by age across health service regions of Western Australia.
**Figure S4.** Patterns of hMPV testing rates by age across health service regions of Western Australia.
**Figure S5.** Number of influenza hospitalisations in children <5 years from PathWest and non–PathWest testing sources in Perth metropolitan region, Western Australia.
**Figure S6.** Positivity rates for respiratory virus by regions of Western Australia.
**Figure S7.** Proportion of laboratory–confirmed RSV hospitalisations for ICD–coded admissions in children <5 years of age by geographical location in the COVID era (2020–2021).

## Data Availability

All data generated or analysed during this study are included in this published article and its supplementary information files. The data within the RSV Data Linkage Platform cannot be shared publicly. Access to the data is subject to approval by relevant data custodians and provided by Data Services at the WA Department of Health (https://www.datalinkage‐wa.org.au/contact‐us/). The use of the data is restricted to named researchers only on the approved ethics protocols.
