# Supplementary material for: Respiratory Viral Testing Rate Patterns in Young Children Attending Tertiary Care Across Western Australia: A Population‐Based Birth Cohort Study
Source: Influenza Other Respir Viruses. 2024 Sep 3;18(9):e70005. doi: 10.1111/irv.70005 (PMC11369639; doi:10.1111/irv.70005)
Supplement: Supplementary file 1 — Table S1. Characteristics of the cohort. Table S2. Respiratory syncytial virus testing rate patterns by age and health regions in Western Australia, 2012–2021. Table S3. Influenza virus testing rate patterns by age and health service regions in Western Australia, 2012–2021. Table S4. Parainfluenza virus testing rate patterns by age and health service regions in Western Australia, 2012–2021. Table S5. Human metapneumovirus testing rate patterns by age and health regions in Western Australia, 2012–2021. Table S6. Respiratory virus positivity rates by age across Western Australia, 2012–2021. Figure S1. Map of Western Australia health regions. Figure S2. Patterns of influenza testing rates by age across health service regions of Western Australia. Figure S3. Patterns of PIV testing rates by age across health service regions of Western Australia. Figure S4. Patterns of hMPV testing rates by age across health service regions of Western Australia. Figure S5. Number of influenza hospitalisations in children <5 years from PathWest and non–PathWest testing sources in Perth metropolitan region, Western Australia. Figure S6. Positivity rates for respiratory virus by regions of Western Australia. Figure S7. Proportion of laboratory–confirmed RSV hospitalisations for ICD–coded admissions in children <5 years of age by geographical location in the COVID era (2020–2021). [file IRV-18-e70005-s001.docx]

**Supplementary Material**

**Respiratory viral testing patterns in young children attending tertiary care across Western Australia: a population-based birth cohort study**

Belaynew W Taye, PhD^*1,2^**,** Mohinder Sarna, PhD^1,2^, Huong Le, PhD^1,2^, Avram Levy, PhD^3,4^, Cara Minney–Smith, MPH^5^, Peter Richmond, FRACP^1,6,7^, Robert Menzies, PhD^8^, Christopher C Blyth, FRACP^1,5,6^, Hannah C Moore, PhD^1,2^

^1^Wesfarmers Centre of Vaccines and Infectious Diseases, Telethon Kids Institute, University of Western Australia, Nedlands, Perth, Australia

^2^School of Population Health, Curtin University, Perth, Australia

^3^Pathogen Genomics and Surveillance Unit, PathWest Laboratory Medicine, QEII Medical Centre, Nedlands, Perth, Australia

^4^School of Biomedical Sciences, University of Western Australia, Nedlands, Perth, Australia

^5^Department of Microbiology, PathWest Laboratory Medicine, QEII Medical Centre, Nedlands, Perth, Australia

^6^School of Medicine, University of Western Australia, Nedlands, Perth, Australia

^7^Department of Paediatric Infectious Diseases, Perth Children’s Hospital, Perth, Australia

^8^Sanofi Vaccines, Australia and New Zealand, Sydney, New South Wales, Macquarie Park, Australia

*Corresponding author:

Belaynew Wasie Taye, MD, MPH, PhD

Wesfarmers Centre of Vaccines and Infectious Diseases

Telethon Kids Institute

University of Western Australia, Western Australia, Perth, Australia

email: [belaynew.taye@telethonkids.org.au](mailto:belaynew.taye@telethonkids.org.au) (Taye BW)

**Table of Contents**

**Table S1** Characteristics of the cohort

**Table S2** Respiratory syncytial virus testing rate patterns by age and health regions in Western Australia, 2012‒2021

**Table S3** Influenza virus testing rate patterns by age and health service regions in Western Australia, 2012‒2021

**Table S4** Parainfluenza virus testing rate patterns by age and health service regions in Western Australia, 2012‒2021

**Table S5** Human metapneumovirus testing rate patterns by age and health regions in Western Australia, 2012‒2021

**Table S6** Respiratory virus positivity rates by age across Western Australia, 2012–2021

**Figure S1:** Map of Western Australia health regions.

**Figure S2** Patterns of influenza testing rates by age across health service regions of Western Australia

**Figure S3** Patterns of PIV testing rates by age across health service regions of Western Australia

**Figure S4** Patterns of hMPV testing rates by age across health service regions of Western Australia

**Figure S5** Number of influenza hospitalisations in children <5 years from PathWest and non–PathWest testing sources in Perth metropolitan region, Western Australia.

**Figure S6** Positivity rates for respiratory virus by regions of Western Australia

**Figure S7** Proportion of laboratory–confirmed RSV hospitalisations for ICD–coded admissions in children <5 years of age by geographical location in the COVID era (2020–2021)

**Table S1 Characteristics of children in the cohort**

|  | Characteristic | | Number of children  N=355,021 |
| --- | --- | --- | --- |
| Age of child | < 12 months | | 288,896 (81.4%) |
|  | 12–23 months | | 37,670 (10.6%) |
|  | 24 – 59 months | | 28,455 (8.0%) |
| Child's sex | Male | | 186,000 (52.5%) |
|  | Female | | 168,043 (47.5%) |
| Gestational age | <32 weeks | | 3,868 (1.1%) |
|  | 32–36 weeks | | 27,104 (7.6%) |
|  | 37 weeks and above | | 324,049 (91.3%) |
| Mother's Ethnicity | Caucasian | | 229,604 (70.7%) |
|  | Aboriginal/Torres Strait Islander | | 18,402 (5.7%) |
|  | Asian | | 45,946 (14.2%) |
|  | African | | 6,181 (1.9%) |
|  | Polynesian | | 5,454 (1.7%) |
|  | Other | | 18,974 (5.8%) |
| WA Health region | Perth metropolitan | | 277,339 (78.1%) |
|  | Southwest | | 23,281 (6.6%) |
|  | Kimberley | | 7,528 (2.1%) |
|  | Pilbara | | 10,518 (3.0%) |
|  | Wheatbelt | | 9,106 (2.6%) |
|  | Goldfields | | 9,625 (2.7%) |
|  | Midwest | | 9,982 (2.8%) |
|  | Great Southern | | 7,642 (2.2%) |
| Remoteness of areas | Major city | | 249,083 (77.1%) |
|  | Inner regional | | 26,002 (8.0%) |
|  | Outer regional | | 24,657 (7.6%) |
|  | Remote |  | 15,343 (4.7%) |
|  | Very remote | | 8,019 (2.5%) |

**Table S2 Respiratory syncytial virus testing rate patterns by age and health regions in Western Australia (2012‒2021)**

| Year of testing | WA Metropolitan Region | | WA Country Region | | | | | | | | | | | | | | | | | | | |
| --- | --- | --- | --- | --- | --- | --- | --- | --- | --- | --- | --- | --- | --- | --- | --- | --- | --- | --- | --- | --- | --- | --- |
|  |  |  | **Southwest** | | **Kimberley** | | **Pilbara** | | | | **Wheatbelt** | | **Goldfields** | | | | | **Midwest** | | **Great Southern** | | |
|  | **Rate** | **95%CI** | **Rate** | **95%CI** | **Rate** | **95%CI** | **Rate** | **95%CI** | | | **Rate** | **95%CI** | | | | **Rate** | **95%CI** | **Rate** | **95%CI** | **Rate** | | **95%CI** |
| <12 months | | | | | | | | | | | | | | | | | | | | | | |
| 2012 | 242.11 | 230.51–254.29 | 105.21 | 82.96–133.44 | 371.89 | 314.93–439.15 | 195.42 | 158.38–241.12 | 164.20 | 127.76–211.04 | | | 364.92 | | 308.84–431.18 | | | 151.17 | 117.12–195.11 | | 100.41 | 69.33–145.43 |
| 2013 | 196.09 | 185.63–207.15 | 103.52 | 82.04–130.63 | 200.44 | 159.60–251.72 | 192.55 | 154.66–239.72 | 168.49 | 131.88–215.26 | | | 265.60 | | 220.33–320.18 | | | 173.44 | 136.01–221.17 | | 178.57 | 132.89–239.96 |
| 2014 | 198.46 | 188.17–209.30 | 99.91 | 79.04–126.28 | 265.43 | 219.42–321.09 | 165.04 | 130.79–208.27 | 208.39 | 166.43–260.92 | | | 299.07 | | 248.51–359.92 | | | 224.05 | 180.21–278.56 | | 130.65 | 90.79–188.00 |
| 2015 | 208.88 | 198.37–219.96 | 130.83 | 106.65–160.49 | 273.59 | 223.99–334.18 | 193.27 | 155.45–240.30 | 261.84 | 210.31–325.98 | | | 355.38 | | 301.31–419.16 | | | 209.04 | 168.35–259.55 | | 146.88 | 103.30–208.86 |
| 2016 | 168.48 | 159.16–178.34 | 122.77 | 99.51–151.48 | 177.44 | 140.15–224.66 | 270.77 | 220.48–332.53 | 141.16 | 102.71–194.00 | | | 507.59 | | 435.96–590.99 | | | 277.63 | 228.22–337.75 | | 218.73 | 167.53–285.59 |
| 2017 | 180.43 | 170.77–190.63 | 123.46 | 99.82–152.71 | 204.79 | 160.29–261.64 | 319.34 | 266.62–382.48 | 144.37 | 104.13–200.14 | | | 517.00 | | 444.44–601.39 | | | 242.75 | 194.98–302.23 | | 239.90 | 185.05–311.01 |
| 2018 | 155.47 | 146.51–164.97 | 212.63 | 179.51–251.86 | 193.83 | 149.51–251.29 | 266.42 | 220.24–322.29 | 134.81 | 95.34–190.63 | | | 686.11 | | 592.85–794.03 | | | 224.13 | 177.90–282.37 | | 169.40 | 124.73–230.07 |
| 2019 | 203.43 | 193.42–213.97 | 242.94 | 209.40–281.86 | 273.47 | 221.10–338.25 | 242.13 | 200.34–292.65 | 143.08 | 102.24–200.25 | | | 725.53 | | 635.91–827.77 | | | 363.85 | 305.35–433.57 | | 236.98 | 182.80–307.23 |
| 2020 | 255.96 | 244.16–268.33 | 194.89 | 161.80–234.73 | 523.76 | 446.06–614.98 | 195.91 | 156.46–245.30 | 132.11 | 91.80–190.11 | | | 396.92 | | 326.59–482.39 | | | 433.66 | 366.57–513.03 | | 171.22 | 121.08–242.11 |
| 2021 | 262.98 | 251.72–274.74 | 335.74 | 293.91–383.52 | 824.34 | 732.13–928.16 | 541.96 | 478.77–613.48 | 151.90 | 110.06–209.65 | | | 504.37 | | 427.12–595.59 | | | 423.19 | 359.22–498.56 | | 442.84 | 370.83–528.82 |
| 12‒23 months | | | | | | | | | | | | | | | | | | | | | | |
| 2012 | 45.99 | 42.44–49.85 | 23.50 | 16.62–33.23 | 80.11 | 59.21–108.40 | 41.47 | 28.82–59.67 | 30.46 | 20.06–46.26 | | | 82.00 | 62.48–107.60 | | | | 40.76 | 27.95–59.43 | | 41.11 | 27.07–62.43 |
| 2013 | 42.26 | 38.85–45.97 | 22.28 | 15.67–31.68 | 92.50 | 69.71–122.74 | 53.02 | 38.07–73.84 | 32.86 | 21.84–49.45 | | | 77.82 | 58.98–102.68 | | | | 30.25 | 19.29–47.42 | | 53.22 | 35.37–80.09 |
| 2014 | 39.24 | 36.05–42.71 | 18.99 | 13.02–27.69 | 82.74 | 61.57–111.18 | 66.92 | 49.97–89.63 | 36.87 | 24.71–55.00 | | | 48.82 | 33.92–70.25 | | | | 41.97 | 28.58–61.64 | | 44.67 | 28.82–69.23 |
| 2015 | 42.96 | 39.63–46.57 | 26.91 | 19.32–37.47 | 85.90 | 63.92–115.43 | 52.49 | 37.69–73.11 | 46.28 | 31.96–67.03 | | | 97.46 | 75.18–126.35 | | | | 41.11 | 27.99–60.38 | | 30.43 | 18.02–51.38 |
| 2016 | 32.63 | 29.76–35.78 | 15.94 | 10.59–23.98 | 62.78 | 44.63–88.31 | 60.55 | 43.27–84.75 | 47.72 | 32.49–70.09 | | | 134.60 | 107.96–167.81 | | | | 53.46 | 38.20–74.81 | | 44.26 | 27.88–70.25 |
| 2017 | 36.06 | 33.10–39.28 | 22.91 | 16.20–32.39 | 71.48 | 50.55–101.08 | 73.40 | 54.63–98.64 | 24.60 | 13.97–43.32 | | | 122.68 | 96.56–155.87 | | | | 48.38 | 33.40–70.07 | | 34.05 | 20.53–56.48 |
| 2018 | 31.51 | 28.73–34.55 | 43.80 | 33.86–56.65 | 76.10 | 53.21–108.84 | 59.53 | 43.49–81.47 | 27.01 | 15.68–46.52 | | | 144.48 | 115.56–180.64 | | | | 66.94 | 48.07–93.24 | | 51.93 | 34.80–77.47 |
| 2019 | 44.80 | 41.51–48.35 | 74.53 | 61.01–91.03 | 111.61 | 84.11–148.10 | 96.80 | 76.59–122.36 | 26.08 | 14.81–45.93 | | | 191.18 | 157.46–232.13 | | | | 104.34 | 80.10–135.90 | | 60.66 | 41.88–87.86 |
| 2020 | 111.42 | 105.91–117.23 | 53.90 | 41.94–69.27 | 272.89 | 225.18–330.72 | 109.00 | 85.94–138.24 | 43.89 | 26.89–71.64 | | | 97.49 | 72.55–131.01 | | | | 213.53 | 176.83–257.86 | | 57.52 | 38.23–86.56 |
| 2021 | 105.43 | 100.27–110.85 | 124.12 | 105.82–145.58 | 457.61 | 400.97–522.26 | 240.43 | 207.32–278.83 | 70.64 | 50.47–98.86 | | | 181.55 | 146.97–224.28 | | | | 148.96 | 120.88–183.58 | | 204.60 | 168.67–248.19 |
| 24‒59 months | | | | | | | | | | | | | | | | | | | | | | |
| 2012 | 24.46 | 20.96–28.55 | 20.41 | 12.51–33.32 | 41.04 | 22.08–76.27 | 44.11 | 26.12–74.48 | 19.46 | 9.28–40.81 | | | 43.87 | 26.45–72.77 | | | | 22.37 | 11.19–44.74 | 13.98 | | 5.25–37.26 |
| 2013 | 18.54 | 16.73–20.55 | 6.31 | 3.74–10.66 | 19.12 | 11.53–31.71 | 17.45 | 10.69–28.49 | 11.73 | 6.66–20.65 | | | 25.90 | 17.36–38.65 | | | | 14.61 | 8.81–24.23 | 14.86 | | 8.23–26.84 |
| 2014 | 14.20 | 12.98–15.54 | 4.11 | 2.52–6.71 | 31.84 | 23.53–43.09 | 14.03 | 9.32–21.11 | 7.54 | 4.38–12.99 | | | 18.07 | 12.11–26.95 | | | | 10.64 | 6.70–16.89 | 8.61 | | 4.63–16.01 |
| 2015 | 14.46 | 13.36–15.66 | 7.86 | 5.67–10.89 | 25.55 | 18.74–34.83 | 20.70 | 15.12–28.33 | 8.80 | 5.47–14.16 | | | 23.56 | 17.00–32.67 | | | | 12.82 | 8.85–18.57 | 12.32 | | 7.77–19.56 |
| 2016 | 10.39 | 9.48–11.39 | 5.04 | 3.35–7.59 | 18.06 | 12.47–26.16 | 20.63 | 14.74–28.88 | 10.60 | 6.84–16.42 | | | 37.93 | 29.45–48.85 | | | | 13.82 | 9.47–20.14 | 14.42 | | 9.40–22.12 |
| 2017 | 10.47 | 9.56–11.46 | 8.35 | 6.08–11.48 | 17.27 | 11.57–25.76 | 19.89 | 14.41–27.45 | 12.46 | 8.04–19.31 | | | 54.64 | 44.23–67.50 | | | | 12.94 | 8.67–19.30 | 15.90 | | 10.66–23.72 |
| 2018 | 10.69 | 9.78–11.68 | 14.10 | 11.08–17.95 | 22.91 | 16.02–32.77 | 19.56 | 14.17–26.99 | 18.00 | 12.59–25.74 | | | 45.34 | 35.69–57.61 | | | | 21.49 | 15.70–29.41 | 16.50 | | 11.06–24.62 |
| 2019 | 14.77 | 13.73–15.90 | 24.70 | 20.53–29.73 | 43.21 | 33.41–55.90 | 39.27 | 31.67–48.69 | 27.71 | 20.69–37.12 | | | 61.94 | 51.02–75.21 | | | | 28.34 | 21.60–37.20 | 24.81 | | 18.06–34.10 |
| 2020 | 34.72 | 33.01–36.51 | 18.25 | 14.51–22.96 | 83.44 | 68.32–101.92 | 22.75 | 16.81–30.78 | 24.12 | 17.15–33.93 | | | 39.23 | 29.97–51.35 | | | | 74.46 | 62.17–89.18 | 21.61 | | 14.82–31.51 |
| 2021 | 38.82 | 37.12–40.60 | 40.21 | 34.88–46.35 | 175.11 | 154.38–198.63 | 110.64 | 97.79–125.18 | 24.87 | 18.38–33.65 | | | 49.83 | 40.02–62.04 | | | | 57.64 | 47.93–69.31 | 80.97 | | 68.27–96.03 |

**Table S3 Influenza virus testing rate patterns by age and health regions in Western Australia (2012‒2021)**

| Year of testing | WA Metropolitan Region | | WA Country Region | | | | | | | | | | | | | | |
| --- | --- | --- | --- | --- | --- | --- | --- | --- | --- | --- | --- | --- | --- | --- | --- | --- | --- |
|  |  |  | **Southwest** | | **Kimberley** | | **Pilbara** | | **Wheatbelt** | | **Goldfields** | | **Midwest** | | **Great Southern** | | |
|  | **Rate** | **95%CI** | **Rate** | **95%CI** | **Rate** | **95%CI** | **Rate** | **95%CI** | **Rate** | **95%CI** | **Rate** | **95%CI** | **Rate** | **95%CI** | **Rate** | **95%CI** |  |
| <12 months | | | | | | | | | | | | | | | | | |
| 2012 | 251.07 | 239.25–263.47 | 105.21 | 82.96–133.44 | 366.54 | 310.02–433.35 | 190.93 | 154.36–236.15 | 172.28 | 134.84–220.11 | 367.56 | 311.27–434.04 | 158.85 | 123.85–203.75 | 86.07 | 57.69–128.41 |  |
| 2013 | 205.77 | 195.04–217.08 | 103.52 | 82.04–130.63 | 200.44 | 159.60–251.72 | 194.95 | 156.80–242.39 | 176.39 | 138.83–224.11 | 265.60 | 220.33–320.18 | 176.11 | 138.36–224.16 | 178.57 | 132.89–239.96 |  |
| 2014 | 209.86 | 199.28–221.01 | 101.34 | 80.31–127.87 | 267.94 | 221.69–323.83 | 165.04 | 130.79–208.27 | 222.10 | 178.63–276.13 | 299.07 | 248.51–359.92 | 226.82 | 182.67–281.63 | 130.65 | 90.79–188.00 |  |
| 2015 | 212.37 | 201.76–223.53 | 135.10 | 110.49–165.19 | 273.59 | 223.99–334.18 | 195.66 | 157.58–242.94 | 261.84 | 210.31–325.98 | 355.38 | 301.31–419.16 | 209.04 | 168.35–259.55 | 142.15 | 99.39–203.30 |  |
| 2016 | 168.34 | 159.03–178.20 | 124.18 | 100.77–153.04 | 177.44 | 140.15–224.66 | 270.77 | 220.48–332.53 | 141.16 | 102.71–194.00 | 507.59 | 435.96–590.99 | 277.63 | 228.22–337.75 | 218.73 | 167.53–285.59 |  |
| 2017 | 180.43 | 170.77–190.63 | 123.46 | 99.82–152.71 | 204.79 | 160.29–261.64 | 319.34 | 266.62–382.48 | 144.37 | 104.13–200.14 | 523.15 | 450.13–608.01 | 242.75 | 194.98–302.23 | 239.90 | 185.05–311.01 |  |
| 2018 | 155.75 | 146.79–165.27 | 212.63 | 179.51–251.86 | 193.83 | 149.51–251.29 | 266.42 | 220.24–322.29 | 134.81 | 95.34–190.63 | 689.92 | 596.39–798.12 | 224.13 | 177.90–282.37 | 169.40 | 124.73–230.07 |  |
| 2019 | 203.57 | 193.55–214.11 | 242.94 | 209.40–281.86 | 273.47 | 221.10–338.25 | 244.40 | 202.39–295.12 | 143.08 | 102.24–200.25 | 725.53 | 635.91–827.77 | 363.85 | 305.35–433.57 | 236.98 | 182.80–307.23 |  |
| 2020 | 256.11 | 244.30–268.49 | 196.64 | 163.40–236.65 | 527.27 | 449.30–618.78 | 195.91 | 156.46–245.30 | 132.11 | 91.80–190.11 | 396.92 | 326.59–482.39 | 433.66 | 366.57–513.02 | 171.22 | 121.08–242.11 |  |
| 2021 | 263.24 | 251.97–275.01 | 345.02 | 302.58–393.41 | 902.85 | 806.10–1011.21 | 541.96 | 478.77–613.48 | 156.00 | 113.52–214.40 | 522.52 | 443.78–615.22 | 426.15 | 361.94–501.76 | 471.88 | 397.35–560.38 |  |
| 12‒23 months | | | | | | | | | | | | | | | | | |
| 2012 | 47.78 | 44.15–51.70 | 23.50 | 16.62–33.23 | 80.11 | 59.21–108.40 | 40.04 | 27.64–57.99 | 30.46 | 20.06–46.26 | 82.00 | 62.48–107.60 | 39.25 | 26.72–57.64 | 39.24 | 25.58–60.18 |  |
| 2013 | 44.60 | 41.09–48.41 | 23.00 | 16.27–32.52 | 92.50 | 69.70–122.74 | 53.02 | 38.07–73.84 | 34.29 | 22.98–51.16 | 77.82 | 58.98–102.68 | 30.25 | 19.29–47.42 | 53.22 | 35.37–80.09 |  |
| 2014 | 41.22 | 37.95–44.77 | 19.70 | 13.60–28.52 | 82.74 | 61.57–111.18 | 71.39 | 53.80–94.73 | 39.94 | 27.19–58.66 | 48.82 | 33.92–70.25 | 43.58 | 29.89–63.55 | 44.67 | 28.82–69.23 |  |
| 2015 | 43.47 | 40.12–47.09 | 27.67 | 19.96–38.37 | 85.90 | 63.92–115.43 | 52.49 | 37.69–73.11 | 46.28 | 31.96–67.03 | 97.46 | 75.18–126.35 | 41.11 | 27.99–60.38 | 30.43 | 18.02–51.38 |  |
| 2016 | 32.56 | 29.69–35.70 | 15.94 | 10.59–23.98 | 62.78 | 44.63–88.31 | 60.55 | 43.27–84.75 | 47.72 | 32.49–70.09 | 134.60 | 107.96–167.81 | 53.46 | 38.20–74.81 | 44.26 | 27.88–70.25 |  |
| 2017 | 36.06 | 33.10–39.28 | 22.91 | 16.20–32.39 | 71.48 | 50.55–101.08 | 73.40 | 54.63–98.64 | 24.60 | 13.97–43.32 | 122.68 | 96.56–155.87 | 48.38 | 33.40–70.07 | 34.05 | 20.53–56.48 |  |
| 2018 | 31.51 | 28.73–34.55 | 43.80 | 33.86–56.65 | 76.10 | 53.21–108.84 | 59.53 | 43.49–81.47 | 27.01 | 15.68–46.52 | 144.48 | 115.56–180.64 | 66.94 | 48.07–93.24 | 51.93 | 34.80–77.47 |  |
| 2019 | 44.87 | 41.58–48.42 | 74.53 | 61.01–91.03 | 111.61 | 84.11–148.10 | 98.19 | 77.81–123.90 | 26.08 | 14.81–45.93 | 191.18 | 157.46–232.13 | 104.34 | 80.10–135.90 | 60.66 | 41.88–87.86 |  |
| 2020 | 111.50 | 105.98–117.30 | 53.02 | 41.16–68.28 | 272.89 | 225.18–330.72 | 109.00 | 85.94–138.24 | 43.89 | 26.89–71.64 | 97.49 | 72.55–131.01 | 215.51 | 178.62–260.02 | 57.52 | 38.23–86.56 |  |
| 2021 | 105.50 | 100.34–110.92 | 124.94 | 106.58–146.47 | 476.33 | 418.47–542.20 | 240.43 | 207.32–278.83 | 72.72 | 52.21–101.28 | 185.78 | 150.75–228.94 | 148.96 | 120.88–183.58 | 208.57 | 172.26–252.54 |  |
| 24‒59 months | | | | | | | | | | | | | | | | | |
| 2012 | 26.13 | 22.50–30.34 | 20.41 | 12.51–33.32 | 41.04 | 22.08–76.27 | 44.11 | 26.12–74.48 | 19.46 | 9.28–40.81 | 46.80 | 28.67–76.39 | 22.37 | 11.19–44.74 | 10.49 | 3.38–32.52 |  |
| 2013 | 20.12 | 18.23–22.21 | 6.31 | 3.74–10.66 | 19.12 | 11.53–31.71 | 17.45 | 10.69–28.49 | 11.73 | 6.66–20.65 | 25.90 | 17.36–38.65 | 14.61 | 8.81–24.23 | 14.86 | 8.23–26.84 |  |
| 2014 | 15.61 | 14.32–17.00 | 4.63 | 2.92–7.35 | 31.84 | 23.53–43.09 | 14.03 | 9.32–21.11 | 8.12 | 4.81–13.71 | 19.57 | 13.33–28.75 | 10.64 | 6.70–16.89 | 8.61 | 4.63–16.01 |  |
| 2015 | 14.75 | 13.63–15.95 | 7.86 | 5.67–10.89 | 25.55 | 18.74–34.83 | 20.70 | 15.12–28.33 | 8.80 | 5.47–14.16 | 23.56 | 17.00–32.67 | 12.82 | 8.85–18.57 | 12.32 | 7.77–19.56 |  |
| 2016 | 10.41 | 9.50–11.42 | 5.04 | 3.35–7.59 | 18.06 | 12.47–26.16 | 20.63 | 14.74–28.88 | 10.60 | 6.84–16.42 | 37.93 | 29.45–48.85 | 13.82 | 9.47–20.14 | 14.42 | 9.40–22.12 |  |
| 2017 | 10.47 | 9.56–11.46 | 8.35 | 6.08–11.48 | 17.27 | 11.57–25.76 | 19.89 | 14.41–27.45 | 12.46 | 8.04–19.31 | 55.27 | 44.80–68.20 | 12.94 | 8.67–19.30 | 15.90 | 10.66–23.72 |  |
| 2018 | 10.69 | 9.78–11.68 | 14.10 | 11.08–17.95 | 22.91 | 16.02–32.77 | 19.56 | 14.17–26.99 | 18.00 | 12.59–25.74 | 45.34 | 35.69–57.61 | 21.49 | 15.70–29.41 | 16.50 | 11.06–24.62 |  |
| 2019 | 14.77 | 13.73–15.90 | 24.92 | 20.73–29.97 | 43.21 | 33.41–55.90 | 39.27 | 31.67–48.69 | 27.71 | 20.69–37.12 | 61.94 | 51.02–75.21 | 28.89 | 22.07–37.81 | 24.81 | 18.06–34.10 |  |
| 2020 | 34.72 | 33.01–36.51 | 18.25 | 14.51–22.96 | 85.18 | 69.88–103.83 | 22.75 | 16.81–30.78 | 24.12 | 17.15–33.93 | 39.97 | 30.61–52.19 | 74.46 | 62.17–89.18 | 21.61 | 14.82–31.51 |  |
| 2021 | 38.88 | 37.18–40.66 | 41.05 | 35.66–47.26 | 177.28 | 156.42–200.93 | 110.64 | 97.79–125.18 | 24.87 | 18.38–33.65 | 51.70 | 41.69–64.11 | 57.64 | 47.93–69.31 | 82.81 | 69.96–98.03 |  |

**Table S4 Parainfluenza virus testing rate patterns by age and health regions in Western Australia (2012‒2021)**

| Year of testing | WA Metropolitan Region | | WA Country Region | | | | | | | | | | | | | | |
| --- | --- | --- | --- | --- | --- | --- | --- | --- | --- | --- | --- | --- | --- | --- | --- | --- | --- |
|  |  |  | **Southwest** | | **Kimberley** | | **Pilbara** | | **Wheatbelt** | | **Goldfields** | | **Midwest** | | **Great Southern** | | |
|  | **Rate*** | **95%CI** | **Rate** | **95%CI** | **Rate** | **95%CI** | **Rate** | **95%CI** | **Rate** | **95%CI** | **Rate** | **95%CI** | **Rate** | **95%CI** | **Rate** | **95%CI** |  |
| <12 months | | | | | | | | | | | | | | | | | |
| 2012 | 241.35 | 229.77–253.51 | 102.12 | 80.23–129.98 | 353.16 | 297.77–418.85 | 188.68 | 152.35–233.67 | 164.20 | 127.76–211.04 | 364.92 | 308.84–431.18 | 151.17 | 117.12–195.11 | 86.07 | 57.69–128.41 |  |
| 2013 | 195.48 | 185.03–206.52 | 102.07 | 80.75–129.01 | 200.44 | 159.60–251.72 | 192.55 | 154.66–239.72 | 168.49 | 131.88–215.26 | 263.19 | 218.14–317.54 | 173.44 | 136.01–221.17 | 178.57 | 132.89–239.96 |  |
| 2014 | 197.87 | 187.60–208.70 | 97.05 | 76.52–123.10 | 262.93 | 217.15–318.35 | 162.72 | 128.74–205.67 | 208.39 | 166.43–260.92 | 296.40 | 246.08–357.00 | 221.28 | 177.74–275.5 | 130.65 | 90.79–188.00 |  |
| 2015 | 208.74 | 198.23–219.81 | 130.83 | 106.65–160.49 | 270.74 | 221.42–331.04 | 193.27 | 155.45–240.3 | 261.84 | 210.31–325.98 | 355.38 | 301.31–419.16 | 209.04 | 168.35–259.55 | 142.15 | 99.39–203.30 |  |
| 2016 | 168.48 | 159.16–178.34 | 121.36 | 98.24–149.92 | 177.44 | 140.15–224.66 | 270.77 | 220.48–332.53 | 141.16 | 102.71–194.00 | 507.59 | 435.96–590.99 | 277.63 | 228.22–337.75 | 218.73 | 167.53–285.59 |  |
| 2017 | 180.43 | 170.77–190.63 | 123.46 | 99.82–152.71 | 201.59 | 157.48–258.05 | 319.34 | 266.62–382.48 | 144.37 | 104.13–200.14 | 517.00 | 444.44–601.39 | 242.75 | 194.98–302.23 | 239.90 | 185.05–311.01 |  |
| 2018 | 155.47 | 146.51–164.97 | 212.63 | 179.51–251.86 | 193.83 | 149.51–251.29 | 263.91 | 217.96–319.54 | 134.81 | 95.34–190.63 | 689.92 | 596.39–798.12 | 224.13 | 177.90–282.37 | 169.40 | 124.73–230.07 |  |
| 2019 | 169.95 | 160.82–179.61 | 241.55 | 208.11–280.36 | 263.82 | 212.47–327.57 | 235.34 | 194.19–285.21 | 117.83 | 81.36–170.66 | 705.83 | 617.51–806.77 | 358.03 | 300.03–427.24 | 224.51 | 171.95–293.14 |  |
| 2020 | 224.93 | 213.89–236.55 | 173.82 | 142.74–211.66 | 516.73 | 439.60–607.39 | 180.44 | 142.76–228.07 | 123.00 | 84.35–179.35 | 381.20 | 312.41–465.14 | 398.58 | 334.49–474.96 | 160.52 | 112.23–229.58 |  |
| 2021 | 204.51 | 194.61–214.91 | 303.25 | 263.63–348.82 | 736.77 | 649.89–835.27 | 485.59 | 425.99–553.53 | 110.85 | 76.02–161.63 | 457.20 | 383.95–544.43 | 384.72 | 323.96–456.88 | 399.28 | 331.22–481.32 |  |
| 12‒23 months | | | | | | | | | | | | | | | | | |
| 2012 | 45.91 | 42.36–49.77 | 22.76 | 16.01–32.37 | 74.39 | 54.35–101.82 | 40.04 | 27.64–57.99 | 29.08 | 18.96–44.59 | 82.00 | 62.48–107.6 | 37.74 | 25.50–55.85 | 39.24 | 25.58–60.18 |  |
| 2013 | 42.26 | 38.85–45.97 | 22.28 | 15.67–31.68 | 92.50 | 69.70–122.74 | 53.02 | 38.07–73.84 | 32.86 | 21.84–49.45 | 77.82 | 58.98–102.68 | 30.25 | 19.29–47.42 | 53.22 | 35.37–80.09 |  |
| 2014 | 39.24 | 36.05–42.71 | 18.99 | 13.02–27.69 | 82.74 | 61.57–111.18 | 68.41 | 51.24–91.33 | 36.87 | 24.71–55.00 | 48.82 | 33.92–70.25 | 41.97 | 28.58–61.64 | 44.67 | 28.82–69.23 |  |
| 2015 | 42.96 | 39.63–46.57 | 26.91 | 19.32–37.47 | 85.90 | 63.92–115.43 | 52.49 | 37.69–73.11 | 46.28 | 31.96–67.03 | 97.46 | 75.18–126.35 | 41.11 | 27.99–60.38 | 30.43 | 18.02–51.38 |  |
| 2016 | 32.56 | 29.69–35.70 | 15.94 | 10.59–23.98 | 62.78 | 44.63–88.31 | 60.55 | 43.27–84.75 | 47.72 | 32.49–70.09 | 134.6 | 107.96–167.81 | 53.46 | 38.20–74.81 | 44.26 | 27.88–70.25 |  |
| 2017 | 36.06 | 33.10–39.28 | 22.91 | 16.20–32.39 | 71.48 | 50.55–101.08 | 73.40 | 54.63–98.64 | 24.60 | 13.97–43.32 | 120.85 | 94.95–153.82 | 48.38 | 33.40–70.07 | 34.05 | 20.53–56.48 |  |
| 2018 | 31.44 | 28.67–34.48 | 43.80 | 33.86–56.65 | 76.10 | 53.21–108.84 | 59.53 | 43.49–81.47 | 27.01 | 15.68–46.52 | 144.48 | 115.56–180.64 | 66.94 | 48.07–93.24 | 51.93 | 34.80–77.47 |  |
| 2019 | 38.36 | 35.33–41.66 | 74.53 | 61.01–91.03 | 109.28 | 82.11–145.45 | 95.42 | 75.37–120.81 | 21.74 | 11.70–40.40 | 189.31 | 155.76–230.07 | 98.65 | 75.17–129.45 | 58.49 | 40.11–85.30 |  |
| 2020 | 95.72 | 90.62–101.11 | 46.83 | 35.78–61.30 | 259.77 | 213.33–316.33 | 105.79 | 83.11–134.65 | 35.66 | 20.71–61.41 | 95.28 | 70.66–128.47 | 199.69 | 164.31–242.70 | 57.52 | 38.23–86.56 |  |
| 2021 | 86.08 | 81.43–91.00 | 111.79 | 94.50–132.25 | 436.81 | 381.55–500.07 | 226.69 | 194.61–264.06 | 64.40 | 45.29–91.58 | 168.89 | 135.65–210.26 | 140.50 | 113.30–174.22 | 190.7 | 156.12–232.93 |  |
| 24‒59 months | | | | | | | | | | | | | | | | | |
| 2012 | 24.31 | 20.82–28.38 | 20.41 | 12.51–33.32 | 36.93 | 19.22–70.98 | 44.11 | 26.12–74.48 | 19.46 | 9.28–40.81 | 43.87 | 26.45–72.77 | 22.37 | 11.19–44.74 | 10.49 | 3.38–32.52 |  |
| 2013 | 18.54 | 16.73–20.55 | 6.31 | 3.74–10.66 | 19.12 | 11.53–31.71 | 17.45 | 10.69–28.49 | 11.73 | 6.66–20.65 | 25.90 | 17.36–38.65 | 14.61 | 8.81–24.23 | 14.86 | 8.23–26.84 |  |
| 2014 | 14.17 | 12.95–15.51 | 4.37 | 2.72–7.03 | 31.09 | 22.89–42.22 | 14.03 | 9.32–21.11 | 7.54 | 4.38–12.99 | 18.07 | 12.11–26.95 | 10.64 | 6.70–16.89 | 8.61 | 4.63–16.01 |  |
| 2015 | 14.44 | 13.34–15.63 | 7.86 | 5.67–10.89 | 25.55 | 18.74–34.83 | 20.70 | 15.12–28.33 | 8.80 | 5.47–14.16 | 23.56 | 17.00–32.67 | 12.82 | 8.85–18.57 | 12.32 | 7.77–19.56 |  |
| 2016 | 10.39 | 9.48–11.39 | 5.04 | 3.35–7.59 | 18.06 | 12.47–26.16 | 20.63 | 14.74–28.88 | 10.60 | 6.84–16.42 | 37.93 | 29.45–48.85 | 13.82 | 9.47–20.14 | 14.42 | 9.40–22.12 |  |
| 2017 | 10.47 | 9.56–11.46 | 8.35 | 6.08–11.48 | 17.27 | 11.57–25.76 | 19.89 | 14.41–27.45 | 12.46 | 8.04–19.31 | 54.64 | 44.23–67.50 | 12.94 | 8.67–19.30 | 15.90 | 10.66–23.72 |  |
| 2018 | 10.69 | 9.78–11.68 | 14.10 | 11.08–17.95 | 22.91 | 16.02–32.77 | 19.56 | 14.17–26.99 | 18.00 | 12.59–25.74 | 45.34 | 35.69–57.61 | 21.49 | 15.70–29.41 | 16.50 | 11.06–24.62 |  |
| 2019 | 12.72 | 11.75–13.77 | 24.26 | 20.13–29.25 | 43.21 | 33.41–55.90 | 38.80 | 31.25–48.17 | 25.86 | 19.11–35.00 | 60.12 | 49.37–73.21 | 27.25 | 20.66–35.96 | 23.51 | 16.96–32.59 |  |
| 2020 | 30.82 | 29.21–32.51 | 16.75 | 13.19–21.29 | 79.10 | 64.41–97.14 | 21.12 | 15.43–28.91 | 22.66 | 15.94–32.22 | 37.75 | 28.69–49.67 | 65.62 | 54.15–79.53 | 21.61 | 14.82–31.51 |  |
| 2021 | 34.01 | 32.42–35.68 | 37.46 | 32.33–43.40 | 172.94 | 152.35–196.32 | 106.25 | 93.67–120.52 | 21.31 | 15.37–29.55 | 42.98 | 33.94–54.41 | 55.09 | 45.62–66.52 | 77.29 | 64.91–92.04 |  |

**Table S5 Human metapneumovirus testing rate patterns by age and health regions in Western Australia (2012‒2021)**

| Year of testing | WA metropolitan Region | | WA regional aeras | | | | | | | | | | | | | | |
| --- | --- | --- | --- | --- | --- | --- | --- | --- | --- | --- | --- | --- | --- | --- | --- | --- | --- |
|  |  |  | **Southwest** | | **Kimberley** | | **Pilbara** | | **Wheatbelt** | | **Goldfields** | | **Midwest** | | **Great Southern** | | |
|  | **Rate** | **95%CI** | **Rate** | **95%CI** | **Rate** | **95%CI** | **Rate** | **95%CI** | **Rate** | **95%CI** | **Rate** | **95%CI** | **Rate** | **95%CI** | **Rate** | **95%CI** |  |
| <12 months | | | | | | | | | | | | | | | | | |
| 2012 | 247.12 | 235.40–259.42 | 105.21 | 82.96–133.44 | 345.13 | 290.43–410.14 | 188.68 | 152.35–233.67 | 166.90 | 130.12–214.07 | 367.56 | 311.27–434.04 | 153.73 | 119.36–197.99 | 82.48 | 54.81–124.12 |  |
| 2013 | 201.47 | 190.85–212.67 | 103.52 | 82.04–130.63 | 200.44 | 159.60–251.72 | 192.55 | 154.66–239.72 | 176.39 | 138.83–224.11 | 265.60 | 220.33–320.18 | 168.10 | 131.32–215.19 | 178.57 | 132.89–239.96 |  |
| 2014 | 204.16 | 193.73–215.16 | 98.48 | 77.78–124.69 | 267.94 | 221.69–323.83 | 162.72 | 128.74–205.67 | 219.35 | 176.19–273.09 | 293.73 | 243.66–354.08 | 224.05 | 180.21–278.56 | 130.65 | 90.79–188.00 |  |
| 2015 | 198.44 | 188.20–209.24 | 129.41 | 105.37–158.93 | 273.59 | 223.99–334.18 | 193.27 | 155.45–240.30 | 255.29 | 204.48–318.72 | 355.38 | 301.31–419.16 | 209.04 | 168.35–259.55 | 137.41 | 95.49–197.73 |  |
| 2016 | 168.34 | 159.03–178.20 | 121.36 | 98.24–149.92 | 177.44 | 140.15–224.66 | 270.77 | 220.48–332.53 | 141.16 | 102.71–194.00 | 507.59 | 435.96–590.99 | 277.63 | 228.22–337.75 | 218.73 | 167.53–285.59 |  |
| 2017 | 180.43 | 170.77–190.63 | 123.46 | 99.82–152.71 | 204.79 | 160.29–261.64 | 319.34 | 266.62–382.48 | 144.37 | 104.13–200.14 | 517.00 | 444.44–601.39 | 242.75 | 194.98–302.23 | 239.90 | 185.05–311.01 |  |
| 2018 | 155.47 | 146.51–164.97 | 212.63 | 179.51–251.86 | 193.83 | 149.51–251.29 | 263.91 | 217.96–319.54 | 134.81 | 95.34–190.63 | 689.92 | 596.39–798.12 | 224.13 | 177.90–282.37 | 169.40 | 124.73–230.07 |  |
| 2019 | 170.36 | 161.22–180.02 | 241.55 | 208.11–280.36 | 267.03 | 215.34–331.13 | 235.34 | 194.19–285.21 | 117.83 | 81.36–170.66 | 715.68 | 626.71–817.27 | 360.94 | 302.69–430.40 | 228.67 | 175.56–297.84 |  |
| 2020 | 224.93 | 213.89–236.55 | 173.82 | 142.74–211.66 | 516.73 | 439.60–607.39 | 180.44 | 142.76–228.07 | 123.00 | 84.35–179.35 | 377.27 | 308.87–460.82 | 398.58 | 334.49–474.96 | 160.52 | 112.23–229.58 |  |
| 2021 | 204.38 | 194.48–214.78 | 303.25 | 263.63–348.82 | 736.77 | 649.89–835.27 | 485.59 | 425.99–553.53 | 110.85 | 76.02–161.63 | 453.57 | 380.64–540.48 | 384.72 | 323.96–456.88 | 399.28 | 331.22–481.32 |  |
| 12‒23 months | | | | | | | | | | | | | | | | | |
| 2012 | 45.84 | 42.29–49.69 | 23.50 | 16.62–33.23 | 74.39 | 54.35–101.82 | 40.04 | 27.64–57.99 | 30.46 | 20.06–46.26 | 82.00 | 62.48–107.60 | 37.74 | 25.50–55.85 | 39.24 | 25.58–60.18 |  |
| 2013 | 42.49 | 39.07–46.22 | 23.00 | 16.27–32.52 | 92.50 | 69.70–122.74 | 53.02 | 38.07–73.84 | 34.29 | 22.98–51.16 | 77.82 | 58.98–102.68 | 30.25 | 19.29–47.42 | 53.22 | 35.37–80.09 |  |
| 2014 | 39.61 | 36.40–43.09 | 18.99 | 13.02–27.69 | 82.74 | 61.57–111.18 | 71.39 | 53.80–94.73 | 38.40 | 25.95–56.83 | 48.82 | 33.92–70.25 | 41.97 | 28.58–61.64 | 44.67 | 28.82–69.23 |  |
| 2015 | 40.34 | 37.12–43.84 | 26.91 | 19.32–37.47 | 85.90 | 63.92–115.43 | 52.49 | 37.69–73.11 | 41.32 | 27.92–61.16 | 97.46 | 75.18–126.35 | 41.11 | 27.99–60.38 | 30.43 | 18.02–51.38 |  |
| 2016 | 32.56 | 29.69–35.70 | 15.94 | 10.59–23.98 | 62.78 | 44.63–88.31 | 60.55 | 43.27–84.75 | 47.72 | 32.49–70.09 | 134.60 | 107.96–167.81 | 53.46 | 38.20–74.81 | 44.26 | 27.88–70.25 |  |
| 2017 | 36.06 | 33.10–39.28 | 22.91 | 16.20–32.39 | 71.48 | 50.55–101.08 | 73.40 | 54.63–98.64 | 24.60 | 13.97–43.32 | 120.85 | 94.95–153.82 | 48.38 | 33.40–70.07 | 34.05 | 20.53–56.48 |  |
| 2018 | 31.44 | 28.67–34.48 | 43.80 | 33.86–56.65 | 76.10 | 53.21–108.84 | 59.53 | 43.49–81.47 | 27.01 | 15.68–46.52 | 144.48 | 115.56–180.64 | 66.94 | 48.07– 93.24 | 51.93 | 34.80–77.47 |  |
| 2019 | 38.57 | 35.52–41.87 | 74.53 | 61.01–91.03 | 111.61 | 84.11–148.10 | 95.42 | 75.37–120.81 | 21.74 | 11.70–40.40 | 189.31 | 155.76–230.07 | 100.54 | 76.81–131.60 | 58.49 | 40.11–85.30 |  |
| 2020 | 95.72 | 90.62–101.11 | 46.83 | 35.78–61.30 | 259.77 | 213.33–316.33 | 105.79 | 83.11–134.65 | 35.66 | 20.71–61.41 | 95.28 | 70.66–128.47 | 199.69 | 164.31–242.70 | 57.52 | 38.23–86.56 |  |
| 2021 | 86.08 | 81.43–91.00 | 111.79 | 94.50–132.25 | 436.81 | 381.55–500.07 | 225.32 | 193.34–262.58 | 64.40 | 45.29–91.58 | 168.89 | 135.65–210.26 | 138.81 | 111.79–172.35 | 190.70 | 156.12–232.93 |  |
| 24‒59 months | | | | | | | | | | | | | | | | | |
| 2012 | 24.92 | 21.38–29.04 | 20.41 | 12.51–33.32 | 36.93 | 19.22–70.98 | 44.11 | 26.12–74.48 | 19.46 | 9.28–40.81 | 46.80 | 28.67–76.39 | 22.37 | 11.19–44.74 | 10.49 | 3.38–32.52 |  |
| 2013 | 19.10 | 17.26–21.14 | 6.31 | 3.74–10.66 | 19.12 | 11.53–31.71 | 17.45 | 10.69–28.49 | 11.73 | 6.66–20.65 | 25.90 | 17.36–38.65 | 14.61 | 8.81–24.23 | 14.86 | 8.23–26.84 |  |
| 2014 | 14.65 | 13.41–16.01 | 4.37 | 2.72–7.03 | 31.09 | 22.89–42.22 | 14.03 | 9.32–21.11 | 7.54 | 4.38–12.99 | 19.57 | 13.33–28.75 | 10.64 | 6.70–16.89 | 8.61 | 4.63–16.01 |  |
| 2015 | 13.73 | 12.65–14.89 | 7.64 | 5.48–10.64 | 25.55 | 18.74–34.83 | 20.70 | 15.12–28.33 | 8.28 | 5.07–13.52 | 23.56 | 17.00–32.67 | 12.36 | 8.48–18.03 | 12.32 | 7.77–19.56 |  |
| 2016 | 10.39 | 9.48–11.39 | 5.04 | 3.35–7.59 | 18.06 | 12.47–26.16 | 20.63 | 14.74–28.88 | 10.60 | 6.84–16.42 | 37.93 | 29.45–48.85 | 13.82 | 9.47–20.14 | 14.42 | 9.40–22.12 |  |
| 2017 | 10.47 | 9.56–11.46 | 8.35 | 6.08–11.48 | 17.27 | 11.57–25.76 | 19.89 | 14.41–27.45 | 12.46 | 8.04–19.31 | 54.64 | 44.23–67.50 | 12.94 | 8.67–19.30 | 15.90 | 10.66–23.72 |  |
| 2018 | 10.69 | 9.78–11.68 | 14.10 | 11.08–17.95 | 22.91 | 16.02–32.77 | 19.56 | 14.17–26.99 | 18.00 | 12.59–25.74 | 45.34 | 35.69–57.61 | 21.49 | 15.70–29.41 | 16.50 | 11.06–24.62 |  |
| 2019 | 12.70 | 11.73–13.75 | 24.26 | 20.13–29.25 | 43.21 | 33.41–55.90 | 38.80 | 31.25–48.17 | 25.86 | 19.11–35.00 | 60.12 | 49.37–73.21 | 27.25 | 20.66–35.96 | 23.51 | 16.96–32.59 |  |
| 2020 | 30.82 | 29.21–32.51 | 16.75 | 13.19–21.29 | 79.10 | 64.41–97.14 | 21.12 | 15.43–28.91 | 22.66 | 15.94–32.22 | 37.75 | 28.69–49.67 | 65.62 | 54.15–79.53 | 21.61 | 14.82–31.51 |  |
| 2021 | 34.03 | 32.44–35.70 | 37.46 | 32.33–43.40 | 172.94 | 152.35–196.32 | 106.25 | 93.67–120.52 | 21.31 | 15.37–29.55 | 42.98 | 33.94–54.41 | 55.09 | 45.62–66.52 | 77.29 | 64.91–92.04 |  |

**Table S6: Respiratory virus positivity rates by age across Western Australia, 2012–2021**

| WA Health region | Respiratory syncytial virus* | | | Influenza virus | | | Parainfluenza virus | | | | Human metapneumovirus | | | |
| --- | --- | --- | --- | --- | --- | --- | --- | --- | --- | --- | --- | --- | --- | --- |
|  | **<12 months** | **12–23 months** | **24–59 months** | **<12 months** | **12–23 months** | **24–59 months** | **<12 months** | **12–23 months** | **24–59 months** | **<12 months** | | **12–23 months** | **24–59 months** |  |
| Perth metropolitan | 26·15 | 20·98 | 15·05 | 1·81 | 3·01 | 6·63 | 5·47 | 7·63 | 5·87 | 4·09 | | 5·39 | 5·73 |  |
| Southwest | 36·69 | 20·32 | 20·74 | 2·04 | 6·24 | 8·98 | 6·85 | 10·84 | 8·58 | 3·91 | | 3·23 | 4·53 |  |
| Kimberley | 25·5 | 18·7 | 14·70 | 5·13 | 8·34 | 14·84 | 7·92 | 10·31 | 10·71 | 4·37 | | 6·01 | 6·76 |  |
| Pilbara | 25·56 | 22·5 | 19·00 | 3·2 | 5·14 | 17·62 | 8·08 | 8·69 | 7·38 | 5·39 | | 4·06 | 4·02 |  |
| Wheatbelt | 23·91 | 17·6 | 13·50 | 2·84 | 6·66 | 4·83 | 4·55 | 8·75 | 13·19 | 3·73 | | 5·46 | 5·55 |  |
| Goldfields | 22·25 | 20·06 | 15·77 | 2·63 | 5·32 | 10·2 | 7·77 | 9·63 | 8·50 | 5·77 | | 6·51 | 5·98 |  |
| Midwest | 26·05 | 18·72 | 19·35 | 3·41 | 7·5 | 9·93 | 6·71 | 7·05 | 8·11 | 3·56 | | 6·11 | 6·22 |  |
| Great Southern | 32·81 | 21·18 | 14·53 | 1·31 | 2·73 | 5·33 | 8·95 | 10·99 | 5·69 | 4·96 | | 7·76 | 6·77 |  |

*Positivity rates reported in percentage


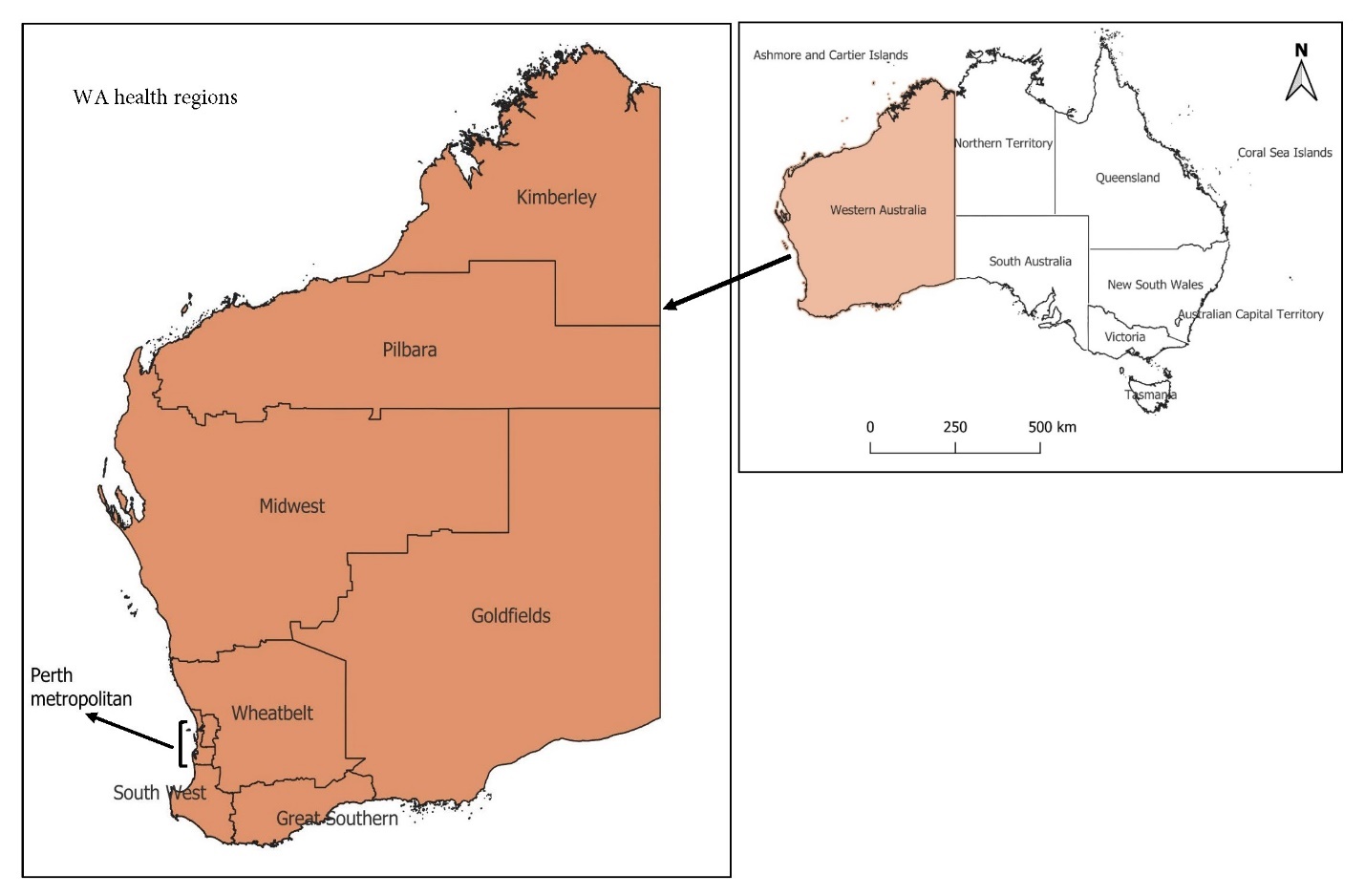


**Figure S1: Map of Western Australia health regions.** Western Australia regional areas include Kimberley, Pilbara, Midwest, Goldfields, Wheatbelt, Southwest and Great Southern. The Perth metropolitan region of Western Australia includes East Metropolitan, North Metropolitan, and South Metropolitan regions of Perth City.


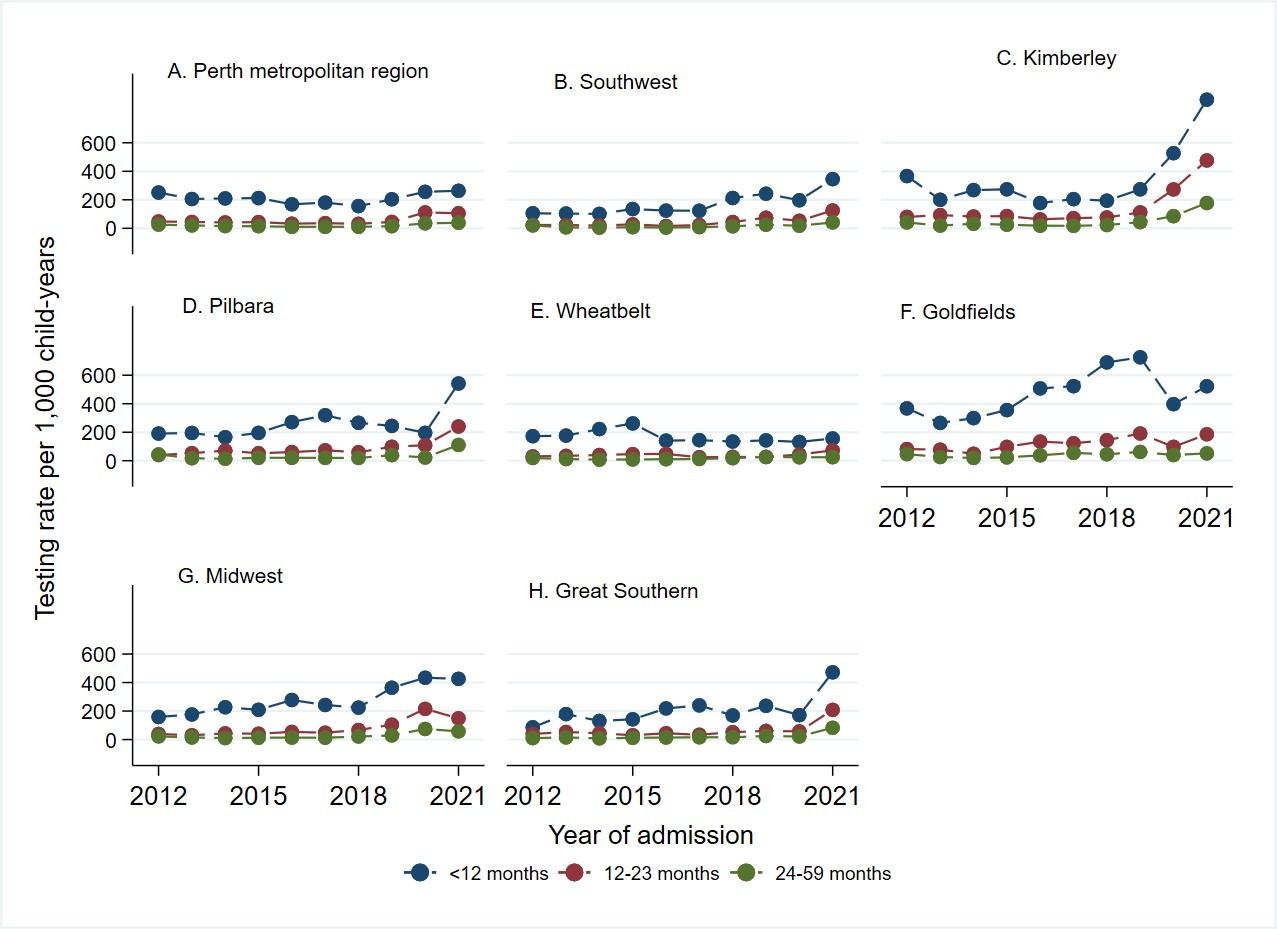


**Figure S2 Pattern of influenza testing by age across health service regions of Western Australia.** The lines present patterns of testing rates for children <12 months (blue), children 12–23 months of age(red), and children 24–59 months of age(green) in Perth metropolitan (A), Southwest(B), Kimberley(C), Pilbara(D), Wheatbelt(E), Goldfields(F), Midwest(G), and Great Southern(H)


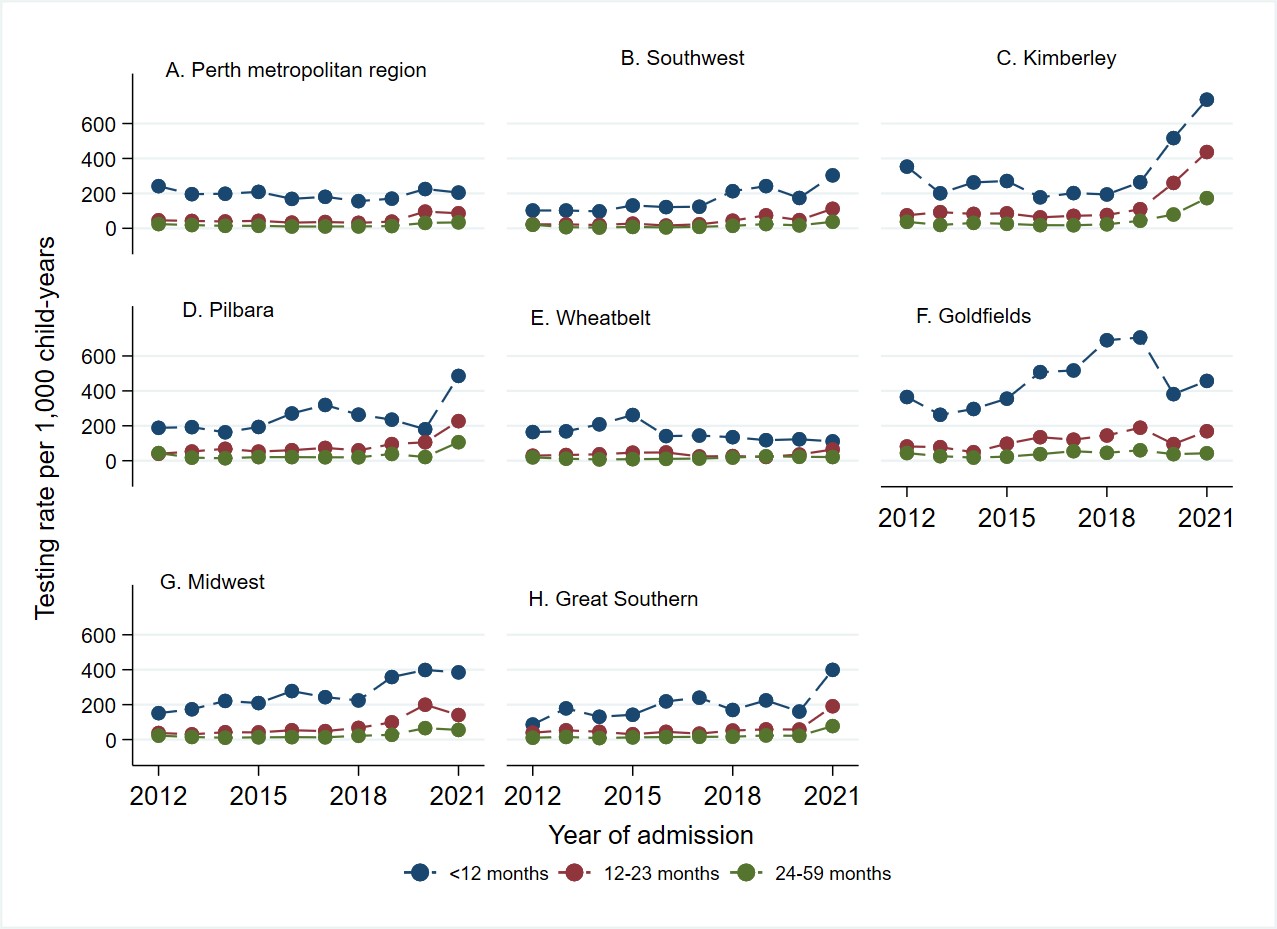


**Figure S3 Patterns of PIV testing by age across health service regions of Western Australia.** The lines present patterns of testing rates for children <12 months (blue), children 12–23 months of age(red), and children 24–59 months of age(green) in Perth metropolitan (A), Southwest(B), Kimberley(C), Pilbara(D), Wheatbelt(E), Goldfields(F), Midwest(G), and Great Southern(H)


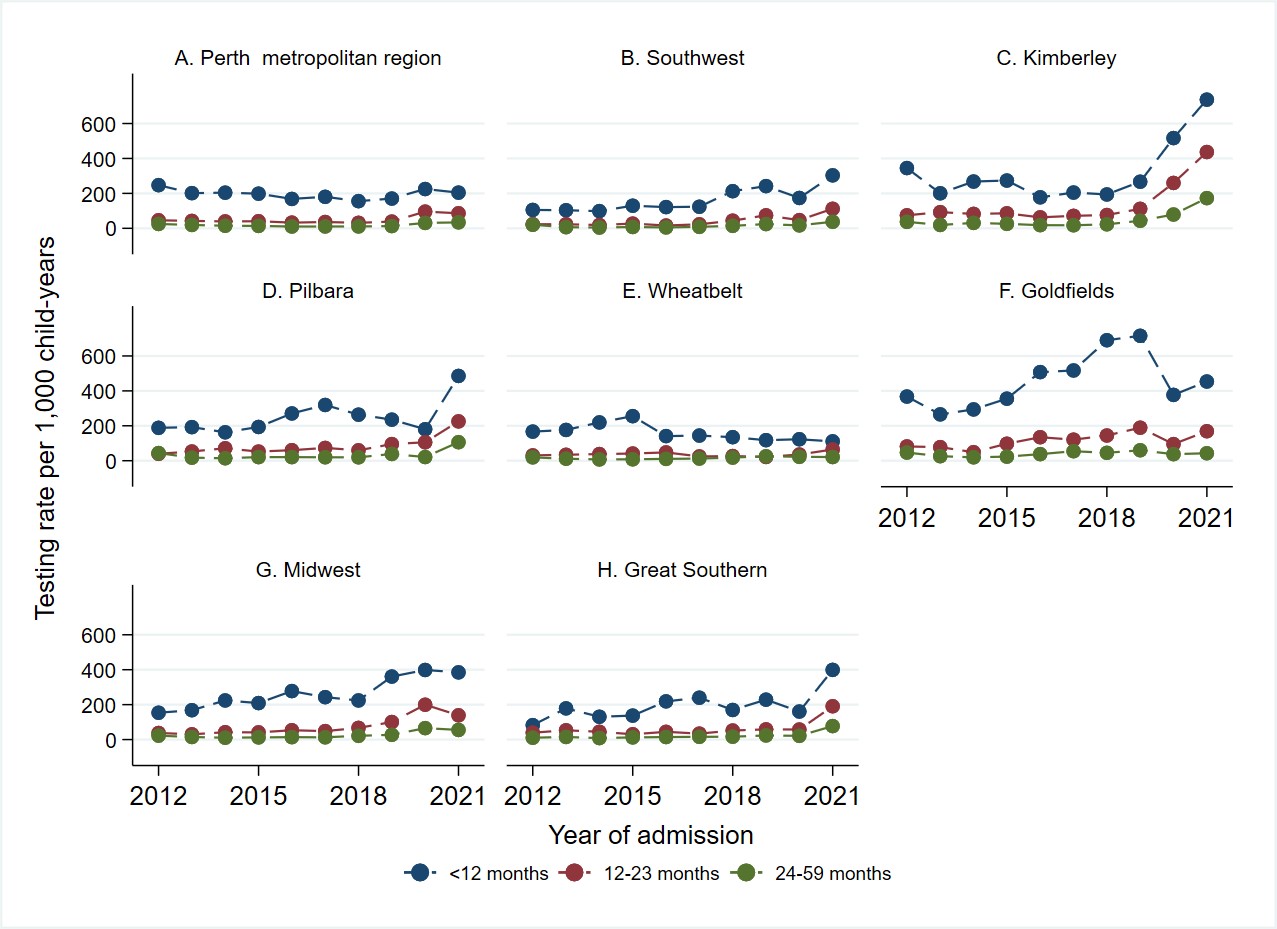


**Figure S4 Patterns of hMPV testing by age across health service regions of Western Australia.** The lines present patterns of testing rates for children <12 months (blue), children 12–23 months of age(red), and children 24–59 months of age(green) in Perth metropolitan (A), Southwest(B), Kimberley(C), Pilbara(D), Wheatbelt(E), Goldfields(F), Midwest(G), and Great Southern(H)


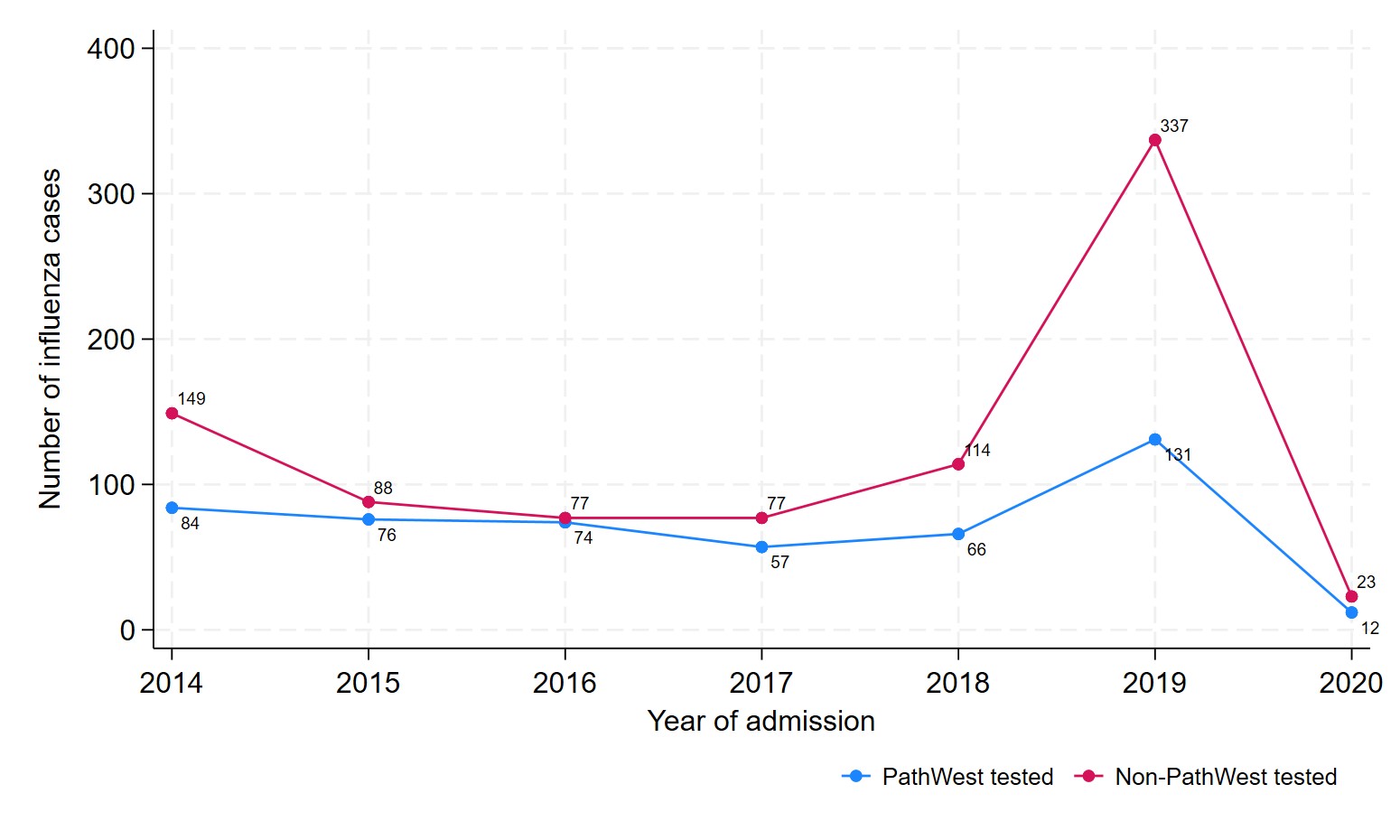
**Figure S5 Number of laboratory–confirmed influenza hospitalisations in children <5 years of age from PathWest Laboratory Medicine and non–PathWest testing sources in Perth metropolitan region, Western Australia.** The blue line represents testing from public microbiological sources, and the red line represents testing from private laboratory sources.


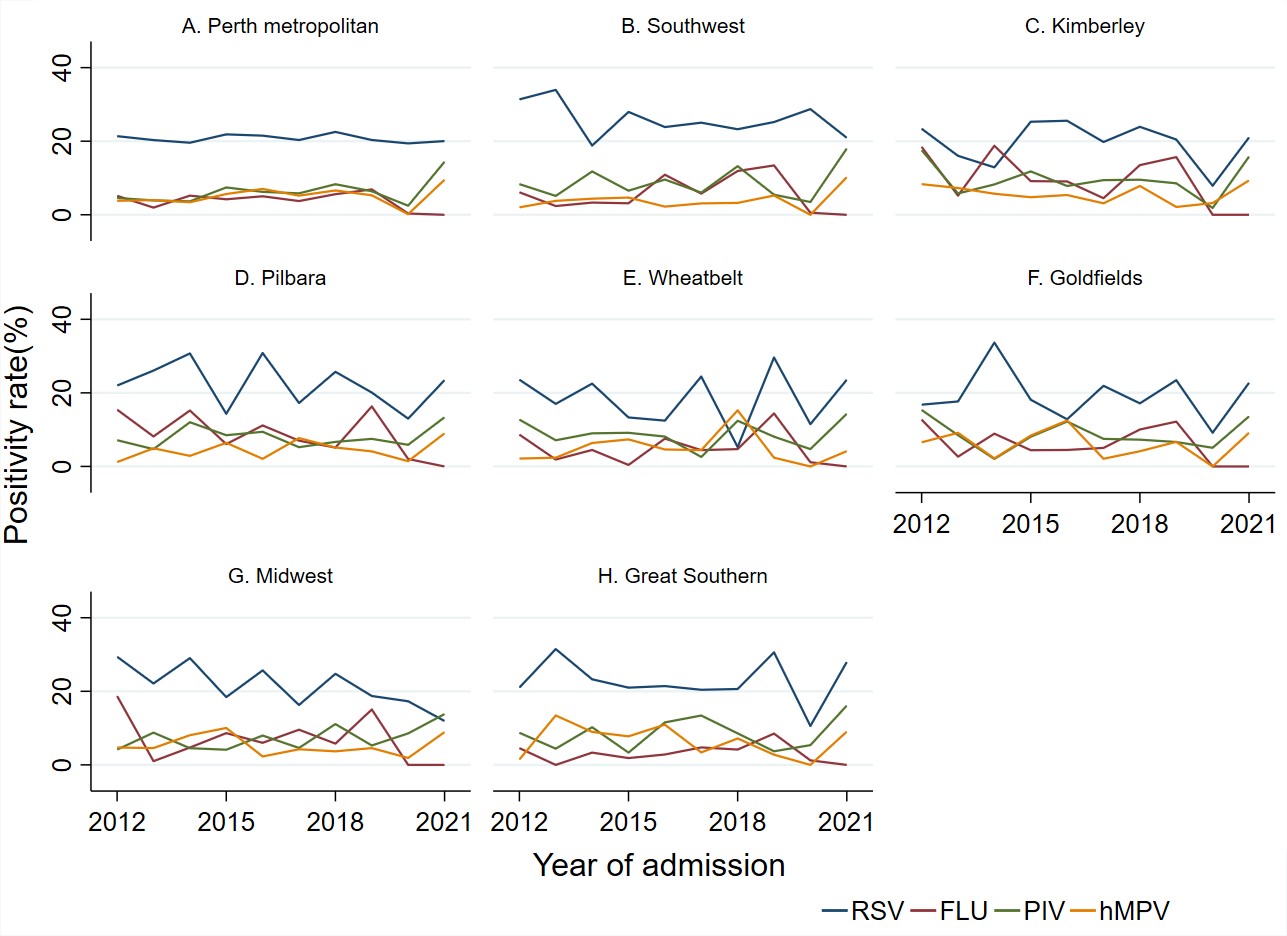
**Figure S6** Respiratory **virus test positivity rate for by year across Western Australia.** The figure includes test positivity rates for RSV (blue), influenza (red), PIV (green), hMPV (orange) in different geographical locations of Western Australia: A. Perth metropolitan region, B. Southwest, C. Kimberley, D. Pilbara, E. Wheatbelt, F. Goldfields, G. Midwest, and H. Great Southern. **WACHS**, Western Australia Country Health Service; **RSV**, respiratory syncytial virus; **Flu**, influenza; **PIV**, parainfluenza virus; **hMPV**, human metapneumovirus.


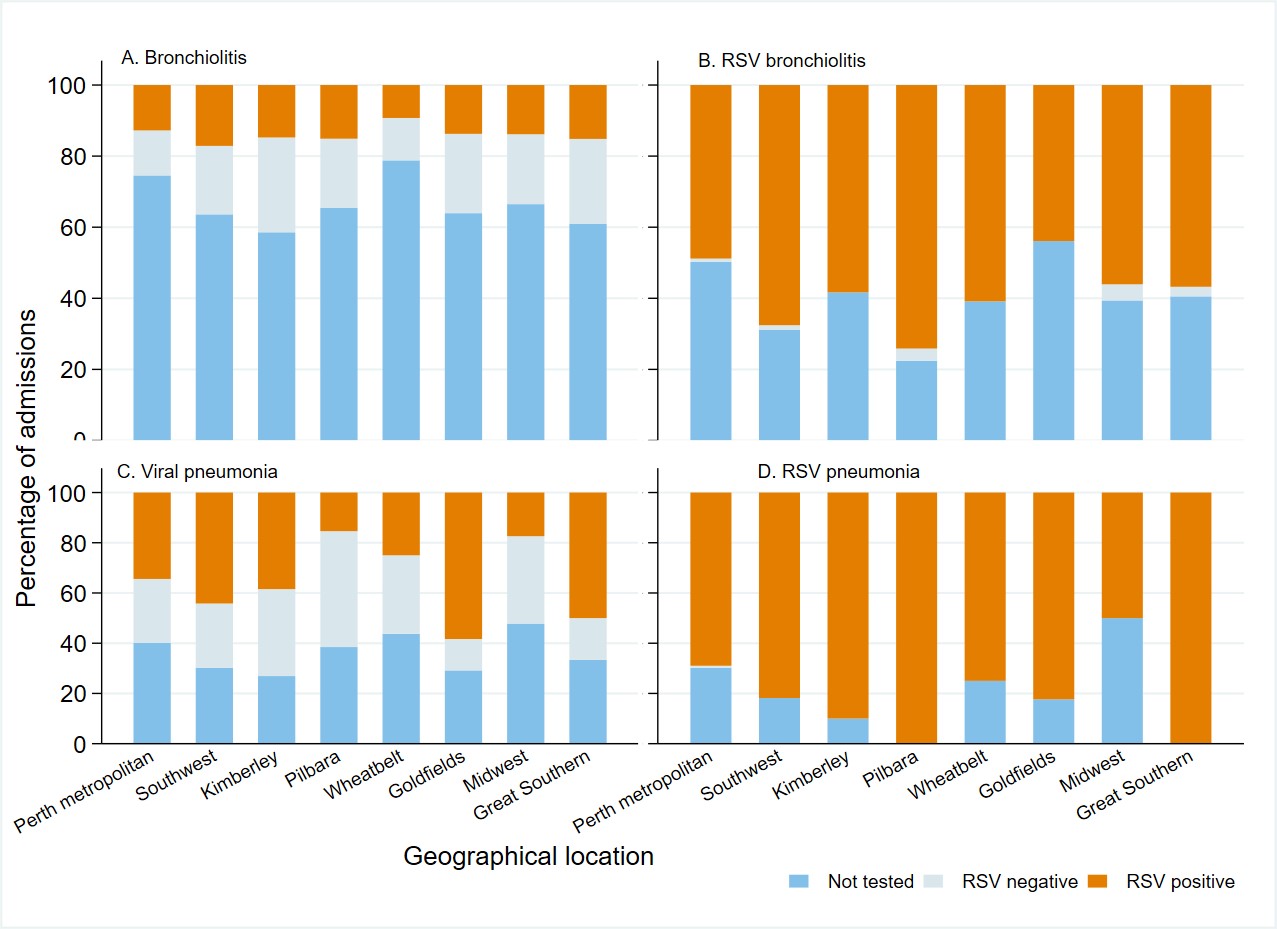


**Figure S7 Proportion of laboratory–confirmed RSV hospitalisations for ICD–coded admissions in children <5 years of age by geographical location in the COVID era (2020–2021).** The figures represent ICD–coded hospitalisations/ED presentations including A) bronchiolitis, B) RSV bronchiolitis, C) Viral pneumonia, D) RSV pneumonia in metropolitan and remote regional areas of WA. **ED**, emergency department; **ICD**, International Classification of Disease; **RSV**, respiratory syncytial virus.
